# Physio-biochemical and molecular stress regulators and their crosstalk for low-temperature stress responses in fruit crops: A review

**DOI:** 10.3389/fpls.2022.1022167

**Published:** 2022-12-12

**Authors:** Amit Kumar Goswami, Naveen Kumar Maurya, Suneha Goswami, Kirti Bardhan, Sanjay Kumar Singh, Jai Prakash, Satyabrata Pradhan, Amarjeet Kumar, Viswanathan Chinnusamy, Prabhat Kumar, Radha Mohan Sharma, Stuti Sharma, Deepak Singh Bisht, Chavlesh Kumar

**Affiliations:** ^1^ Division of Fruits and Horticultural Technology, ICAR-Indian Agricultural Research Institute, New Delhi, India; ^2^ Division of Biochemistry, ICAR-Indian Agricultural Research Institute, New Delhi, India; ^3^ Department of Basic Sciences and Humanities, Navsari Agricultural University, Navsari, India; ^4^ Multi Testing Technology Centre and Vocational Training Centre, Selesih, Central Agricultural University, Imphal, India; ^5^ Division of Plant Physiology, ICAR-Indian Agricultural Research Institute, New Delhi, India; ^6^ Department of Agriculture and Farmers Welfare, Ministry of Agriculture & Farmers Welfare, Govt. of India, Krishi Bhavan, New Delhi, India; ^7^ Department of Plant Breeding and Genetics, Jawaharlal Nehru Krishi Vishwavidyalaya, Jabalpur, Madhya Pradesh, India; ^8^ ICAR-National Institute for Plant Biotechnology, New Delhi, India

**Keywords:** cold tolerance, transcriptome, aquaporins, membrane injury, phytohormone, CBFs

## Abstract

Low-temperature stress (LTS) drastically affects vegetative and reproductive growth in fruit crops leading to a gross reduction in the yield and loss in product quality. Among the fruit crops, temperate fruits, during the period of evolution, have developed the mechanism of tolerance, i.e., adaptive capability to chilling and freezing when exposed to LTS. However, tropical and sub-tropical fruit crops are most vulnerable to LTS. As a result, fruit crops respond to LTS by inducing the expression of LTS related genes, which is for climatic acclimatization. The activation of the stress-responsive gene leads to changes in physiological and biochemical mechanisms such as photosynthesis, chlorophyll biosynthesis, respiration, membrane composition changes, alteration in protein synthesis, increased antioxidant activity, altered levels of metabolites, and signaling pathways that enhance their tolerance/resistance and alleviate the damage caused due to LTS and chilling injury. The gene induction mechanism has been investigated extensively in the model crop Arabidopsis and several winter kinds of cereal. The ICE1 (inducer of C-repeat binding factor expression 1) and the CBF (C-repeat binding factor) transcriptional cascade are involved in transcriptional control. The functions of various CBFs and aquaporin genes were well studied in crop plants and their role in multiple stresses including cold stresses is deciphered. In addition, tissue nutrients and plant growth regulators like ABA, ethylene, jasmonic acid etc., also play a significant role in alleviating the LTS and chilling injury in fruit crops. However, these physiological, biochemical and molecular understanding of LTS tolerance/resistance are restricted to few of the temperate and tropical fruit crops. Therefore, a better understanding of cold tolerance’s underlying physio-biochemical and molecular components in fruit crops is required under open and simulated LTS. The understanding of LTS tolerance/resistance mechanism will lay the foundation for tailoring the novel fruit genotypes for successful crop production under erratic weather conditions.

## Introduction

**Figure 1:**
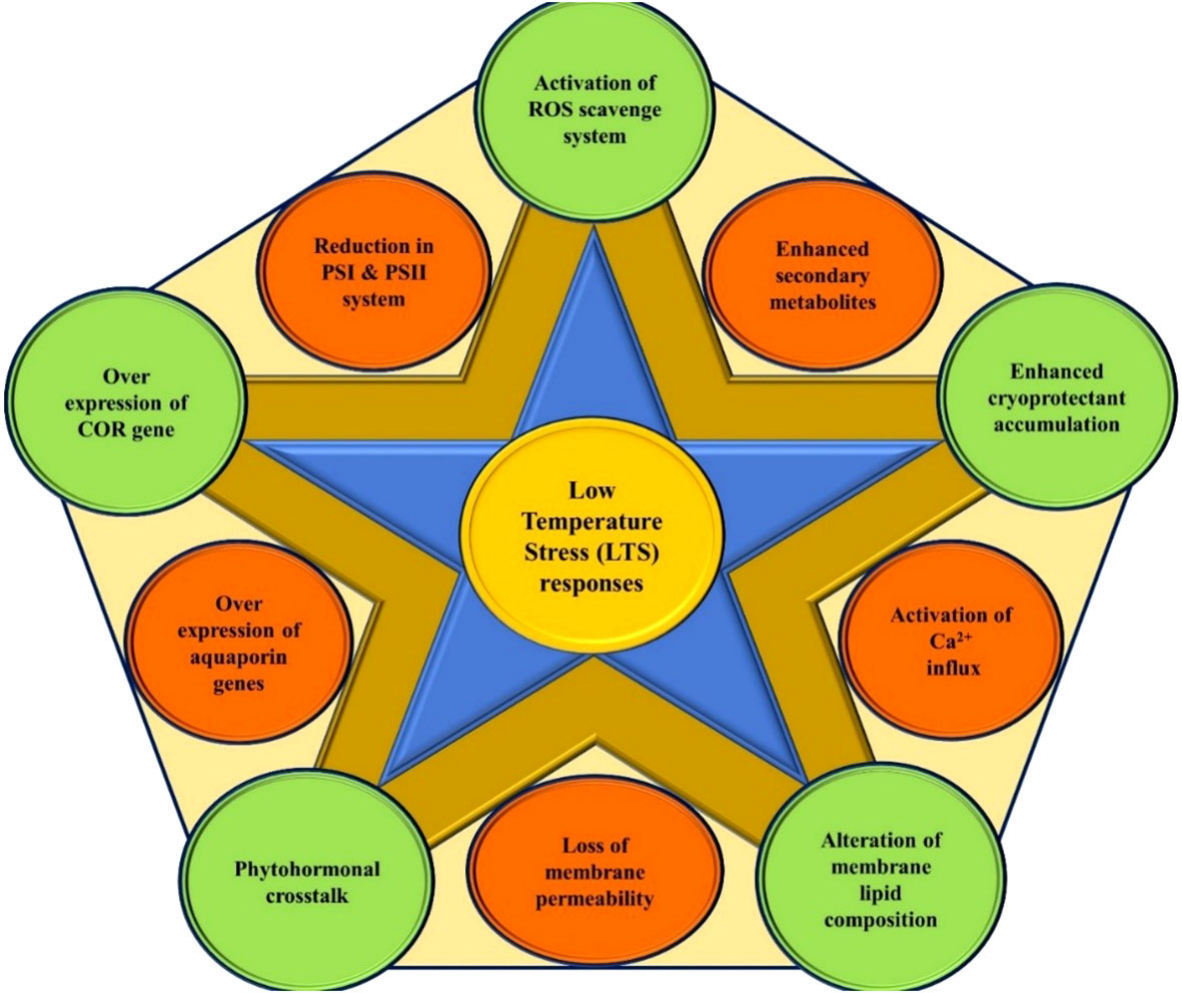
Low-temperature stress responses in plant system.

Plants are sessile and experience several abiotic stresses episode in their life due to harsh climatic conditions. These circumstances triggered several molecular changes in the plants, which led to a variety of responses of physiology and biochemistry of the plant cells, which determine the fitness of the plant in the environment. Temperature is one of the decisive and essential abiotic factors regulating plant’s ontogeny. The process for how Plants sense temperature is elusive. However, several candidate thermos-sensors proteins are known that respond to the membrane changes and other cellular changes triggered by low temperature, leading to downstream events of change in the pattern of gene expressions and plant’s responses (Ding and Yang, 2022). Low temperature (LT) influences plant growth and development by causing various morpho-physiological and biochemical changes (Nishiyama, 1976; Medina et al., 2011) (**Figure 1**). LT has an impact on many processes, including, photosynthesis, cell division, membrane stability, absorption and transport of water and nutrients, yield, and ultimately species survival. Plants experience low temperature stress (LTS) in two ways: (i) Chilling stress: when plants are exposed to LT below 10–15°C for a certain period, causing injury without formation of ice crystals within the plant cells; (ii) Freezing stress: when plants are exposed to sub-zero °C low temperatures, inducing ice crystallization of cellular content, resulting in cell dehydration and freezing injury (Beck et al., 2007; Zhu et al., 2007). Two processes lead to freezing injury: (i) vitrification, which results from the fast freezing of cellular substance, and (ii) supercooling, which results in the formation of ice crystals from intra or extracellular content. An overview of the optimum temperature range for vegetative and reproductive growth for some tropical and subtropical fruit species is given in **Table 1**. Eco-physiological and biophysical studies on tropical and subtropical fruit species are crucial for understanding how LTS affects fruit crops and how it might be managed. (Barlow et al., 1974). Tropical and subtropical fruit species exhibit distinct damaging symptoms when exposed to LT below 10–15°C for a certain period, resulting in drastic yield reduction or even death over prolonged exposure (Chen and Patterson, 1985; Herner, 1990; Alonso et al., 1997). The ability to withstand mild non-freezing temperature stress has evolved over thousands of years in temperate fruits such as apple, pear, peach, plum, strawberry, *etc*. The coping mechanism used by temperate fruits to adjust to cold conditions comprises alterations in a number of physio-morphological and biochemical parameters as well as the expression of stress-associated genes (Medina et al., 2011).

**Table 1:** Optimum temperature ranges for the growth of some tropical and subtropical fruit species.

Sl. No.	Fruit crop	Optimum temperature (°C)	Reference (s)
01	Grapevine *(Vitis vinifera)*	10-35°C	White et al. (2006)
02	Banana *(Musa* spp.*)*	20-35°C	Viktorova (1983)
03	Guava *(Psidium guajava)*	23-28°C	Verheij and Coronel (1992)
04	Mango *(Mangifera indica)*	24-27°C	Davanport (2009)
05	Logan *(Dimocarpus longan)*	20-25°C	Verheij and Coronel (1992)
06	Rambutan (*Nephelium lappaceum*)	25-32°C	Verheij and Coronel (1992)
07	Jackfruit *(Artocarpus heterophyllus)*	16-28°C	Haq (2006)
08	Litchi (*Litchi chinensis)*	25-35°C	Tindall (1994)
09	Durian *(Durio zibethinus)*	24-30°C	Verheij and Coronel (1992)
10	Langsat (*Lansium domesticum* L.)	25-35°C	UDPSM (2002)
11	Pomelo (*Citrus maxima* L.)	23-30°C	Verheij and Coronel (1992)
12	Cashew nut *(Anacardium occidentale)*	20-35°C	Joubert and Thomas (1965)
13	Coconut *(Cocos nucifera)*	20-32°C	Louis and Annappan (1980)
14	Mangosteen (*Garcinia mangostana* L.)	25-35°C	Osman and Milan (2006)
15	Citrus (*Citrus* spp.)	23-27°C	Verheij and Coronel (1992)

Temperate fruit crops undergo dormancy during the winter to avoid the detrimental consequences of LTS. Different physio-biochemical changes are brought on by LTS exposure in tolerant and sensitive genotypes. The extent of changes decides the genotype’s tolerance and ability to acclimate to the cold stress (Barlow et al., 1974; Martin and Douglas, 1979). The aim of this review is to understand physio-morphological, biochemical, and molecular processes connected to LTS in fruit crops as well as the role of phytohormones and other signaling molecules in reducing low-temperature damage. A better understanding of the physiological mechanisms underlying LTS tolerance in fruit crops will relate to improved crop damage management and breeding strategies to combat LTS.

## Physiological responses

LTS significantly affects the vegetative and reproductive growth of tropical and subtropical fruits (Alonso et al., 1997), thereby supressing their yield. For instance, if the temperature falls <4°C in the winter for over three hours, the papaya plants may die due to the leakage of white (oozing out milky) latex from the stem of frost-damaged plants (Ram, 2005). Frost or unexpected temperature fluctuations during late winter cause severe damage to fruit crop foliage, crown, flowers, and fruits resulting in a limited yield of low-quality papaya fruit, which ripens unevenly (Singh et al., 2010). Moreover, under chilling temperatures and humid conditions, a hermaphrodite flower of papaya may revert to femaleness (carpelloidy of stamens), resulting in deformed fruits (Awada, 1958; Storey, 1969; Ram, 2005; Lin et al., 2016). In coffee plants, temperatures <16°C suppress vegetative growth, limiting net photosynthesis, resulting in irregular maturity and poor yields (Bauer et al., 1985). However, the reduction in vegetative growth was not due to the decrease in leaf water potential but it was attributed to more prolonged exposure to LT (<16°C exposure) (Barros et al., 1997). However, during LTS acclimation, relative water content (RWC) of the leaves in guava was associated with decrease in vegetative growth. The non-acclimated guava leaves had three to four time’s higher anthocyanin accumulation than the latter cold-acclimated guava leaves (Hao et al., 2009). Similarly, Pradhan et al. (2017) reported that under the LT regimes, RWC and fresh leaf weight in papaya decreased, while dry leaf weight reflected the opposite trend. Pradhan et al. (2018) found that the percent change in plant height in control papaya plants was more substantial than its LT-treated counterpart. The decrease in plant growth was due to the reduced photosynthetic rate under LT. In addition, chilling stress in plant roots can alter their metabolic heat rates as detected by micro calorimetry (Criddle et al., 1988), cause cortical destruction and reduce root elongation (Harrington and Kihara, 1960).

### Cellular changes

Several studies have found that cell membrane networks are the primary sites of freezing injury in plants (Levitt, 1980; Steponkus, 1984), and that freeze-induced membrane damage is caused primarily by the acute dehydration caused by freezing (Steponkus, 1984; Steponkus, 1993). The extracellular fluids of the apoplastic space contain a lower solute concentration than the intracellular fluid and thus have a higher freezing point; therefore, ice formation is initiated first in the apoplastic space (Jan and Andrabi, 2009). Since ice has a lower water potential than liquid, extracellular ice has a lower water potential than inside the cell, resulting in dehydration. LTS causes some cellular function abnormalities, and membrane damage and the LTS injury is evaluated through electrolyte leakage, changes in membrane lipid composition, and malondialdehyde (MDA) production. Cell dehydration leads to lipid peroxidation and an increase in MDA content, as well as damage to cell membrane fluidity, thus disrupting membrane selectivity, resulting in the permeability of unwanted nutrient elements and ions, leading to ion leakage and disrupting cellular ionic homeostasis (Mahajan and Tuteja, 2005; Yadav, 2010; Shin et al., 2018). The membrane stability index (MSI) of papaya genotypes exposed to LT regimes decreased (Pradhan et al., 2019) while the membrane injury index (MII) increased (Pradhan et al., 2017). Cold stress reduced the MSI gradually, indicating a loss of cell membrane integrity. Campos et al. (2003) reported that electrolyte leakage and lipid degradation influence cold sensitivity in *Coffea* sp. leaves.

Numerous studies have noted that chilling stress significantly reduces the photosynthetic efficiency of sensitive plants (Yang et al., 2005; Fariduddin et al., 2011). The disruption of the thylakoid ETS pathway and carbon reduction cycle significantly altered photosynthesis under LTS. Furthermore, stomatal control of CO_2_ supply may be primarily responsible for decreasing the net photosynthetic rate (Allen and Ort, 2001). Limiting stomatal conductance may be attributed to loss of turgor of guard cells due to LTS induced dehydration. The ultrastructure of chloroplasts has changed after a prolonged cooling period, and thus ability to intercept light energy may be lost (Yang et al., 2005). Potassium (K) and calcium (Ca) were two essential nutrients in improving plant chilling tolerance. Several studies have revealed that electrolyte leakage is primarily related to K^+^ efflux from plant cells, mediated by plasma membrane cation conductance. When K+ levels are low, photo-oxidative damage caused by chilling or frost is worsened. This makes plant growth and yield decrease.

High cellular potassium levels can protect against oxidative damage caused by cooling or frost (Waraich et al., 2012). Furthermore, several studies have shown that applying a higher concentration of K+ could reduce LTS-induced damage in crops such as potatoes (Grewal and Singh, 1980), carnations (Kafkafi, 1990), and vegetable seedlings (Hakerlerler et al., 1997). Pradhan et al. (2018) also reported a high level of K^+^ content with a decline in stomatal conductance and transpiration rate, which would help to maintain plant water status. Recently, Maurya et al. (2020) also noted an increase in leaf K^+^ content in papaya seedlings grown under LTS. Similarly, Ca also influences both development and responses to environmental challenges like cold stress by regulating various physiological processes in plants at the tissue, cellular, and molecular levels (Waraich et al., 2011). Plant genotypes that are LTS tolerant can maintain their high leaf water potential by closing their stomata and limiting water loss during transpiration (Wilkinson et al., 2001). However, Yadav (2010) reported that dehydration occurs under exposure to LTS due to low water uptake due to stomata closure.

Furthermore, Ca also modulates the stress responses during cold injury, healing, and adaptation to cold stress (Palta, 1990). LTS activates Ca^2+^channels, resulting in the accumulation of Ca^2+^ in the cytosol and the induction of phospholipid signaling. The vacuole is the intracellular source of Ca^2+^, and which triggers stomatal closure with increasing Ca^2+^ levels in the cell, a distinguishing feature of plants grown at or exposed to LTS. According to some studies, Ca^2+^ (released from internal guard cell stores or the apoplast) regulates ABA-induced stomatal closure (Wilkinson et al., 2001). Ca^2+^ is required for LTS recovery because it activates the plasma membrane enzyme ATPase, which is needed to pump nutrients lost during the cell injury back into the cell (Palta, 1990). Calcium also acts as calmodulin under LTS conditions, regulating plant metabolic activity and promoting plant development (Waraich et al., 2012). Compared with different *Carica papaya* genotypes, Pradhan et al. (2018) found the highest Ca^2+^accumulation in the leaf and roots of a cold-tolerant wild relative of papaya, *V. cundinamarcensis.*

### Chlorophyll

The most critical component of the photosystem is chlorophyll. LTS represses chlorophyll bio-synthesis and accumulation in actively growing leaves (Glaszmann et al., 1990). For example, the chlorophyll content of papaya seedlings at a 20°/10°C (day/night) temperature regime was 8.16% lower than the control plants at 28°/18°C (day/night). Cold-tolerant genotypes may accumulate higher chlorophyll content under LTS than cold-sensitive lines (Pradhan et al., 2019). An alternative measure for determining the freezing tolerance and accessing freezing damage and cold acclimation responses of leaves is chlorophyll fluorescence (Ehlert and Hincha, 2008). The chlorophyll fluorescence technique has been employed in various other crops to assess the extent of photodamage at LT, including *Arabidopsis* (Ehlert and Hincha, 2008), soybean (Tambussi et al., 2004), maize (Aroca et al., 2001), *etc*. Regardless of the plant species investigated, Maxwell and Johnson (2000) found that the value of chlorophyll fluorescence (*Fv*/*Fm*) in healthy leaves was close to 0.80. The lower value of chlorophyll fluorescence (*Fv*/*Fm*) indicates photoinhibition of PS II reaction canters, disrupted due to LTS. Smillie (1979) observed that the quantum efficiency (QE) of the PS II center (*Fv*/*Fm*) in papaya was 0.42 in the winter season, having a temperature range of 6°/17°C (min./max.) and 0.72 in the summer, in a temperature range of 18°/26°C (min./max.), indicating that LT likely lowered the PSII activity. Perturbance of photosynthetic machinery in Strawberry, also reflected by chlorophyll fluorescence ((*Fv/Fm*) and LTS significantly reduced the chlorophyll fluorescence (*Fv/Fm*) value (Zareei et al., 2021). Pradhan et al. (2019) also found reduction in *Fv/Fm* during LTS. The treated plants expressed a significantly lower *Fv*/*Fm* value than the control. The lowest observed values were 0.438 and 0.584 in the temperature regime of 20°/10°C (day/night), followed by 22°/12°C (day/night), respectively. In contrast, the control plants (28°/18°C day/night) exhibited the highest (0.736) value of *Fv*/*Fm*. Classified mango cultivars based on LTS and *Fv*/*Fm* responses. An *Fv/Fm* ratio of less than 0.5 is denoted as chilling sensitive, while those with an *Fv*/*Fm* value greater than 0.6 are considered as chilling tolerant cultivars.

### Photosynthesis

With the lowering of temperature, the rate of metabolic processes slows steadily and eventually stops under extreme stress (Taiz and Zeiger, 2002). LT affects different dimensions of photosynthesis in fruit crops. Cellular photosynthesis under LTS is substantially inhibited or reduced due to the disruption of all major components, including the thylakoid electron transport system and carbon reduction cycle. After a long cooling period, the chloroplast ultrastructure has altered, and the capacity to efficiently trap light energy may be lost by the thylakoids membranes (Yang et al., 2005). LTS thermodynamically reduces enzymatic activities, such as Calvin cycle enzymes and ROS (Reactive Oxygen Species) scavenging enzyme activities. LT causes a photostatic imbalance in thylakoid membranes, leading to an over-reduction of the electron transport chain and ROS generation at PSII and PSI (Soitamo et al., 2008; Ruelland et al., 2009; Yun et al., 2010). Papaya plants exposed to an LT regime of 20°/10°C (day/night) exhibited a 57.96% reduction in the photosynthesis rate compared to the control plants. This reduction was genotype-dependent and decided their degree of tolerance. The chilling susceptible Red Lady papaya had a drastic reduction (82.10%) compared to the other genotypes (Pradhan et al., 2018). Similarly, Grau and Halloy (1997) also reported a 15% reduction in the photosynthesis rate of papaya plants exposed to the LT regime (15°/5°C; day/night; 4 days) as compared to the control (25°/15°C; day/night). LTS also contributes to ROS generation by reducing the leaf gas exchange in fruit crops due to decreased stomatal conductance. The genotypes that can withstand LTS can keep their leaf water potential high by closing their stomata and avoiding water loss through transpiration. (Wilkinson et al., 2001). In mango, reported that LTS was attributed to a rise in stomatal resistance of net CO_2_ assimilation rate and a decrease in Rubisco activity, chlorophyll concentrations, *etc*., with increased activity of the chlorophyll-degrading enzyme chlorophyllase. Similarly, among various papaya genotypes, the cold-tolerant wild relative *V. cundinamarcensis* exhibited a lower transpiration rate than other susceptible genotypes (Pradhan et al., 2018). Grau and Halloy (1997) also reported that the reduction in stomatal conductance under cold stress was up to 25-30% compared to the control in papaya.

## Biochemical responses

The biochemistry of various cellular components and reactions changed during LTS to help the plant acclimatize to the cold. LTS induced changes range from changes in membrane lipids composition to cellular metabolites, disruption in the equilibrium of gel to liquid-crystalline phase, synthesis of diverse cryoprotective molecules *viz.* soluble sugars (raffinose, saccharose, stachyose, trehalose), sugar alcohols (inositol, ribitol, sorbitol) and low-molecular-weight nitrogenous compounds (glycine betaine, proline) (Janska et al., 2009).

### Cell membrane

Phospholipids are the fundamental components of the plasma membrane, and their composition and properties give membrane fluidity and selectivity. The cell membrane is the first place affected by LTS damage, which results in alterations in the plasma membrane’s lipid composition. Compared to non-acclimated plants, the amount of unsaturated fatty acids in the cellular membranes of acclimated plants increases, minimizing injury caused by threshold temperature (Theocharis et al., 2012). These modifications protect the plasma membrane and chloroplast envelope from LTS injury (Matteucci et al., 2011). However, in susceptible genotypes, the proportion of saturated fatty acids in the phosphatidyl glycerol (PG) backbone of the chloroplast membrane was higher under the LTS. (Yokoi et al., 1998).

The ROS produced under LTS due to photostatic imbalance induced lipid peroxidation in the cell, damaging the cell membrane’s structural and dynamic features, and hence responsible for the non-functionality of the membrane by modifying the fluidity, ion leakage, and producing malondialdehyde (MDA). For example, lipid order in a phospholipid bilayer of a cell membrane diminishes when lipid peroxidation products are added. Pore formation might occur if all phospholipids are oxidized. Reactive species such as reactive oxygen and nitrogen species (RONS) will be able to cause oxidative damage to intracellular macromolecules like DNA and proteins, too (Alonso et al., 1997; Van der Paal et al., 2016). Coffee seedlings, while exposed for six days at the different treatments of temperatures (10°, 15°, 20°, and 25°C), a higher amount of MDA is generated under low temperatures (10°C), leading to higher electrolyte leakage from the root tip. Under completely controlled conditions. Pradhan et al. (2019) evaluate the effects of five LT regimes on five *Carica papaya* genotypes and one distantly related cold-tolerant plant, *V. cundinamarcensis*, They observed lower MDA content in the cold-tolerant papaya genotype and *V. cundinamarcensis* than in the other *C. papaya* genotypes. The higher rate of oxidation of membrane lipids in susceptible genotypes was linked to a higher amount of MDA, which caused damage to the membrane.

### LTS related proteins

LTS affects a variety of metabolites and metabolic processes, including lipoproteins. Cryoprotection, which is the change of lipid biosynthetic enzymes during LTS (Miura and Furumoto, 2013), keeps the plasma membrane, chloroplastic envelope, and other cellular membranes safe and stable. Plant cells synthesize the specific proteins under the LTS, primarily hydrophilic in nature. Cold-regulated proteins (CORs), Low-Temperature Induced (LTI), Late Embryogenesis Abundant (LEA), Responsive to Abscisic acid (RAB), Early Responsive to Dehydration (ERD), Cold Induced (KIN), heat-shock proteins (HSPs), *etc*. are examples of such protein families. Dehydrins are the most common LEA proteins during cold acclimation and stabilize cell membranes against FI (Bies-Etheve et al., 2008; Sun et al., 2013). Hao et al. (2009) performed the leaf protein analyses after cold acclimation in guava and observed the accumulation of dehydrin protein in FI tolerant guava variety (Lucknow-49). Several freeze-induced membrane injuries have been associated with ice nucleation in the apoplast initiated by freezing. It has also been reported that denaturation of proteins occurs in plants under LT, potentially resulting in cellular damage (Guy et al., 1985). For instance, Pradhan et al. (2017) noted a 35.51% higher total soluble protein content in the cold-treated papaya leaves than in the non-treated plants. The cold-tolerant wild relative, *V. cundinamarcensis*, reported the highest increase (79.06%), while the lowest was in the cold susceptible variety, Pusa Nanha (8.78%).

### Antioxidant

**Figure 2:**
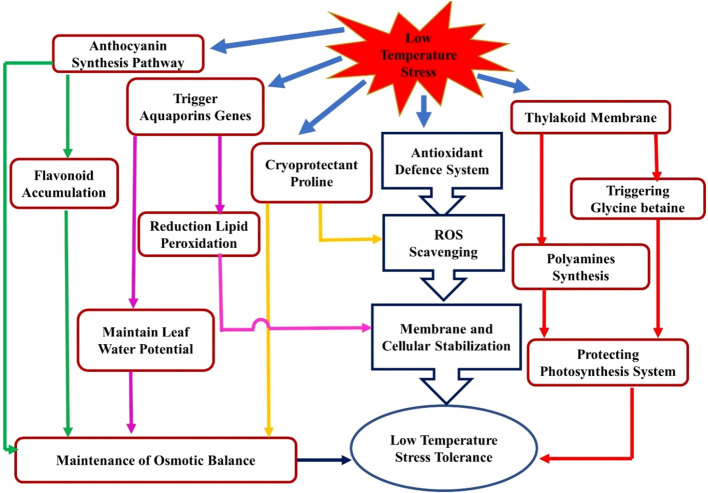
Systematic representation of stress responses and stress regulator crosstalk for low-temperature tolerance in plants.

Low temperature induced membrane injuries also accelerate generation of reactive oxygen species (ROS), and enhanced antioxidants activities plays key role in cold acclimation. (Arora, 2018). Antioxidant enzymes influence plant abiotic stress tolerance as they play a crucial role in balancing the ROS concentration within the cell. The antioxidant defense system of plants is comprised of non-enzymatic antioxidants such as glutathione, vitamin C (ascorbic acid), phenolic compounds, flavonoids, carotenoids, α-tocopherol, the osmolyte proline, and antioxidant enzymes such as CAT (catalase), PPO (polyphenol-oxidase), SOD (superoxide dismutase), POD (peroxidase), GR (glutathione reductase), DHAR (dehydro ascorbate reductase), MDHAR (monodehydro ascorbate reductase), and APOX (ascorbate peroxidase) (Cao et al., 2009; Kumar and Yadav, 2009; Zhang et al., 2009 and Ding and Wang, 2018). Geng et al. (2019) reported that overexpression of antioxidant enzymes such as CAT, POX, and SOD under cold stress dramatically lowers the ROS levels in *Citrus grandis*. Lee and Lee (2000) also reported increasing antioxidant enzyme activity in cucumbers under chilling stress. The LT induces CAT and SOD, which act as antioxidant system and are integral parts of the plant’s defensive responses under LTS (**Figure 2**). It is evident that *V. cundinamarcensis*, a cold-tolerant wild relative of papaya, also expressed higher SOD, GPX, APX, and GR (Pradhan et al., 2017). However, SOD and APX activities increased in cultivated papaya regardless of genotype under LT conditions (Maurya et al., 2020). In strawberries, the increased levels of H_2_O_2_ and MDA are associated with decreased activity of SOD, POD, and CAT under LTS (Xu et al., 2020).

Non enzymatic antioxidants also play a crucial role in ROS balancing during abiotic stresses, including LTS (Ahmad et al., 2010). For instance, higher lycopene containing peels of grapes show better ROS scavenging, and higher chilling tolerance, without significant effect of enzymatic antioxidants activities (Lado et al., 2016). Though, Rooy et al. (2017) also observed cold tolerance of grapevines was associated with both, carotenoids as well as enzymatic antioxidants activities. In cold sensitive, mandarin, chilling injury was associated with low carotenoid content (Rey et al., 2020). However, effective antioxidant defense against ROS, is dependent upon the coordinate action of enzymatic and non-enzymatic antioxidants (Kerchev and Van Breusegem, 2022). For instance, activities of GR, DHAR, MDHAR and APX regulate glutathione-ascorbic acid (GSH/AA) cycle (Foyer and Noctor, 2011) and thus exogenous application of compounds which boosts the plants antioxidants defense, ameliorate the chilling injury in fruits (Zhang et al., 2021).

### Cryoprotectant

Accumulation of compatible osmolytes or osmoprotectants in the cytoplasm is a typical response under various abiotic stresses, where dehydration is involved, including LTS. For instance, plants exposed to chilling stress accumulated more fructans and had better freezing tolerance (Livingston et al., 2009). They safeguard the structure of proteins and enzymes; help to maintain the osmotic gradient between the cell’s surroundings and the cytoplasm (Kempf and Bremer, 1998; Fedotova, 2019). Under cold stress, the organic osmolytes like sugars, proline, glycine betaine, amino acids, *etc*., change the cell’s osmotic potential. This change narrows the water potential gradient between the apoplastic region of ice generation and the cytoplasm. As a result, the pace at which water moves away from the cell slows down, and the cell membrane becomes more stable, making it more resistant to LTS stress (Elbein et al., 2003). The role of osmoprotectants in ameliorating LTS is well known in fruit crops. Red Delicious apple’s cortical tissues and buds’ cold hardiness were associated with the concentration of total sugars, sorbitol, and RFO (raffinose family oligosaccharides) (Stushnoff et al., 1993). Another compatible solute, trehalose (α-D-glucopyranosyl-1, 1-α-D- glucopyranoside), a non-reducing disaccharide, is found in many organisms, including bacteria, fungi, insects, and plants. One of the crucial functions of trehalose is to maintain biological membranes and proteins through H-bonding during dehydration due to FI (Donnamaria et al., 1994; Goddijn and Van Dun, 1999; Elbein et al., 2003). Plants exposed to chilling stress accumulated more fructans and had better freezing tolerance (Livingston et al., 2009).

Proline, a type of amino acid, is found abundantly in higher plants and is known to accumulate in considerable amounts in response to environmental challenges (Hayat et al., 2012; Jogawat, 2019). Proline helps maintain osmotic balance and helps in scavenging free radicals (**Figure 2**) and their crosstalk. It also serves to preserve the integrity of subcellular structures (e.g., membranes and proteins), limits electrolyte leakage, and buffers cellular redox potential during stress by bringing ROS concentrations into normal ranges (Ozturk and Demir, 2002; Hsu et al., 2003; Kishor et al., 2005). There is a significant correlation between cold stress tolerance and increased proline content in various plants (Swaaij et al., 1985; Dorffling et al., 1990; Kaplan et al., 2007). Kushad and Yelenosky (1987) observed the effect of proline and polyamines in LTS tolerance in three citrus genotypes: sour orange (SO), *Citrus aurantium* L., ‘Valencia’ (VAL), *Citrus sinensis* (L.) Osbeck, and rough lemon (RL), *Citrus jambhiri* Lush. After three weeks of exposure to cold hardening (temperature range: 15.6°/4.4°C day/night) and non-hardening (temperature range: 32.2°/21.1°C day/night), Sour orange had the highest proline content (7.9mg/g dry weight), whereas the lowest content (4.2 and 3.9mg/g dry weight) was found in VAL and RL, respectively. According to the authors, there is a direct association between higher spermidine (Spd) content and citrus cold hardiness. Pradhan et al. (2019) also found a significant proline accumulation in papaya genotypes as temperatures decrease.

Strawberry (*Fragaria* x *ananassa* Duch.) crown sugar content was also positively correlated with cold tolerance (Paquin et al., 1989). The amount of sucrose decreased, and total sugar content in the crowns of Bounty and Redcoat cultivars increased during cold hardening and peaking in January, coinciding with maximum hardiness. The high glucose levels, fructose, raffinose, and stachyose content are attributed to cold hardiness in Chardonnay and Riesling grape (*Vitis vinifera*) buds and cortical tissues (Hamman et al., 1996). An endophytic gram-negative bacteria (*Burkholderia phytofirmans)* altered carbohydrate metabolism in the grapevine (*V. vinifera*) and helped to acclimatize to LTS (Fernandez et al., 2012). Most sugars, such as mannose, glucose, fructose, sucrose, galactinol, raffinose, and maltose, were higher when grapevine was treated with *B. phytofirmans* under LTS. Yooyongwech et al. (2009) found that the high-chill peach cv. Kansuke Hakuto had a higher total soluble sugar content in the bud cushion sample during December and January (temperature about 2°C), but the low-chill peach cv. Coral had a lower total soluble sugar content. Pradhan et al. (2019) found that cold (20°/10°C; day/night) exposed papaya plants had 117.10 percent higher total soluble sugars than control plants. Polyamines, such as putrescine (Put), spermidine spermidine: (Spd), and spermine (Spm), play a significant role in the green mango (*Mangifera indica* L. ‘Kensington Pride’) LT tolerance during storage (Nair and Singh, 2004). Similarly, membrane integrity and fluidity were protected from LTS, in pomegranates due to higher polyamine levels during storage (Mirdehghan et al., 2007). In addition, experimental evidence suggests that polyamines also play a role in protecting photosynthetic function (**Figure 2**). Polyamines interact with proteins present in the thylakoid membranes, particularly the light-harvesting complex II (LHCII) and the PSII, through hydrogen bonding and stabilize the tertiary structure of proteins under stress (Kotzabasis et al., 1993; Hamdani et al., 2011). Glycine betaine is an ammonium molecule synthesized in chloroplasts that accumulates under stress conditions, including high salt levels and cold temperatures (Sakamoto and Murata, 2002). In four weeks of cold-acclimation treatment of strawberry (*Fragaria* x *ananassa* Duch.), LTS tolerance was raised from -5.8° to -17°C with a two-fold increase in endogenous glycine betaine levels in the leaves (Rajashekar et al., 1999). Exogenous glycine betaine (2 mM) to unhardened plants boosted cold leaf tolerance nearly two-fold within 72 h. Furthermore, it promoted whole-plant freezing survival and regeneration. Sirooeinejad et al. (2020) investigated the level of freezing tolerance of four pomegranate (*Punica granatum* L.) varieties, including ‘Alak Torsh’ (AT), ‘Tabestaneh Torsh’ (TT), ‘Poostsefid Torsh’ (PT), and ‘Poostsyah Shirin’ (PS), and its association with several physiological events and antioxidant response during cold acclimation and de-acclimation. The lethal temperature (LT_50_) of pomegranate shoots was positively associated with MDA, starch, and antioxidant enzyme activity but negatively related to total phenolic content, proline, and soluble carbohydrates. Similarly, Pradhan et al. (2019) also noted a significant positive correlation of LT exposed papaya plantlet survival (%) with relative water content (RWC), membrane stability index (MSI), and chlorophyll fluorescence, while noting a significant negative association with MDA, proline, and sugar contents.

### Aquaporins

Aquaporins are membrane-bound water channels that facilitate water flow with small solutes across the membrane (Moshelion et al., 2015). Plant aquaporins are present in the plasma membrane and other cellular membranes like the tonoplast, endoplasmic reticulum, plastids, and membrane compartments that interact with symbiotic organisms in some species (Baiges et al., 2002; Maurel et al., 2015). Aquaporins are classified into four clades: AQP1, AQP2, AQP3, and AQP4, of a broad superfamily of major intrinsic proteins (MIPs). Various reports show that aquaporins play a significant role in imparting abiotic stress tolerance, including LTS tolerance (Yooyongwech et al., 2009; Lee et al., 2012; Li et al., 2015; Wei et al., 2019) in various crops. Several reports have suggested that aquaporins play a significant role in the cold tolerance of temperate fruits during winter dormancy. Yooyongwech et al. (2009) reported that gamma tonoplast intrinsic protein (*Pp-γTIP1*) and plasma membrane intrinsic protein (*Pp-PIP1)* play essential roles in intra- and intercellular membrane transport, enhancing cold resistance in the bud cushions of high-chill peach cultivars.

Among the tropical fruits, the role of aquaporins was studied in cold tolerance in bananas. According to Sreedharan et al. (2013), transgenic banana plants that overexpressed a natural plasma membrane aquaporin (*MusaPIP1;2*) showed remarkable tolerance to cold stress. In addition, transgenic lines showed lower MDA accumulation, higher proline content, RWC, and increased photosynthetic efficiency (*Fv*/*Fm*) than controls under exposure to cold stress. Moreover, these transgenic plants exhibited lower damage to the cell membrane and chloroplastic membrane under LTS. Cold-tolerance of banana Dajiao, also associated with aquaporins *viz.* MaPIP1;1, MaPIP1;2, MaPIP2;4, MaPIP2;6, MaTIP1;3, and membrane bounds Peroxidase 52 and Peroxidase P7. These proteins are the vital cellular adaptations that led to a reduction in lipid peroxidation and maintained leaf cell water potential (WP) (**Figure 2**) (He et al., 2018). Xu et al. (2020) reported that the overexpression of *MaPIP2-7*, an aquaporin gene in bananas, improved tolerance to various stresses, including cold, salt, and drought. When compared to wild-type (WT) banana plants under stress, *MaPIP2-7* transgenic plants had reduced levels of MDA and ion leakage (IL) but higher levels of chlorophyll content, proline, soluble sugar, and abscisic acid (ABA). Furthermore, Xu et al. (2021) determined the function of the aquaporin gene MaPIP1;1 and confirmed that overexpression led to multiple stress tolerance in bananas, including drought and cold. Recently, Lopez-Zaplana et al. (2022) elucidated that aquaporins (*PIP2;1*, *PIP2;5* and *PIP2;6*) decreases with the magnitude of different abiotic stresses in melon.

## Signaling and molecular responses

### Phytohormones

Plant growth-regulating hormones such as abscisic acid (ABA), ethylene, jasmonic acid, and salicylic acid (SA) play a significant role during abiotic stresses, including cold, salinity, drought, light, and heavy metal stresses and they acts as links between stress regulator and responses at cellular, tissue and organ level to external stimuli (Rachappanavar et al., 2022). When *Vitis vinifera* cv. ‘Pinot Gris’ was sprayed with ABA @ 400 mg L^-1^ between veraison and three weeks post veraison, it efficiently accelerated and induced deeper dormancy and increased bud frost tolerance without influencing vine size, yield, or fruit quality (Li et al., 2015). Postharvest foliar treatments of ABA @ 5000 ppm to Merlot grapevines promoted leaf abscission and boosted autumn bud cold tolerance. However, ABA at a lower concentration of 1000 ppm encourages a delay in bud break in the spring season. It has been noted that after ABA treatments, fruit yield and elemental composition were less affected (Bowen et al., 2016). Foliar application of ABA (500 mg· L^-1^) increased freeze tolerance in grapefruit trees (Wang et al., 2020). In grapevine, ethylene is released under low-temperature stress, which induces the expression of ethylene-responsive factor (*VaERF057*) and enhances cold tolerance (Sun et al., 2016). Ethylene plays a crucial role in preventing chilling injury in nectarines (Zhou et al., 2001). In *Arabidopsis*, cold stress reduces the amount of active gibberellin (GA) content and the accumulation of DELLAs, which are negative regulators of GA (Achard et al., 2006; Achard et al., 2016). SA treatment (2 mM) is highly effective in reducing the chilling injury effects, electrolyte leakage, and ascorbic acid loss in the husk of pomegranates (Sayyari et al., 2009). Furthermore, SA treatment alleviates chilling injury in ‘Qingnai’ plum fruit during postharvest storage (Luo et al., 2011). Exogenous application of methyl jasmonate (MeJA) (10^-4^ M) reduced the chilling injury index and ion leakage in Tommy Atkins mangos during storage at 7°C (Gonzalez-Aguilar et al., 2000). Application of MeJA vapor for 20 h at 20°C to ‘Kent’ mango fruits has enhanced the shelf-life under cold storage with a lower chilling injury score (Gonzalez-Aguilar et al., 2001). Similar reports are also available for guava (Gonzalez-Aguilar et al., 2004), loquat (Cao et al., 2009), and pomegranate (Sayyari et al., 2011).

### Molecular responses

**Figure 3:**
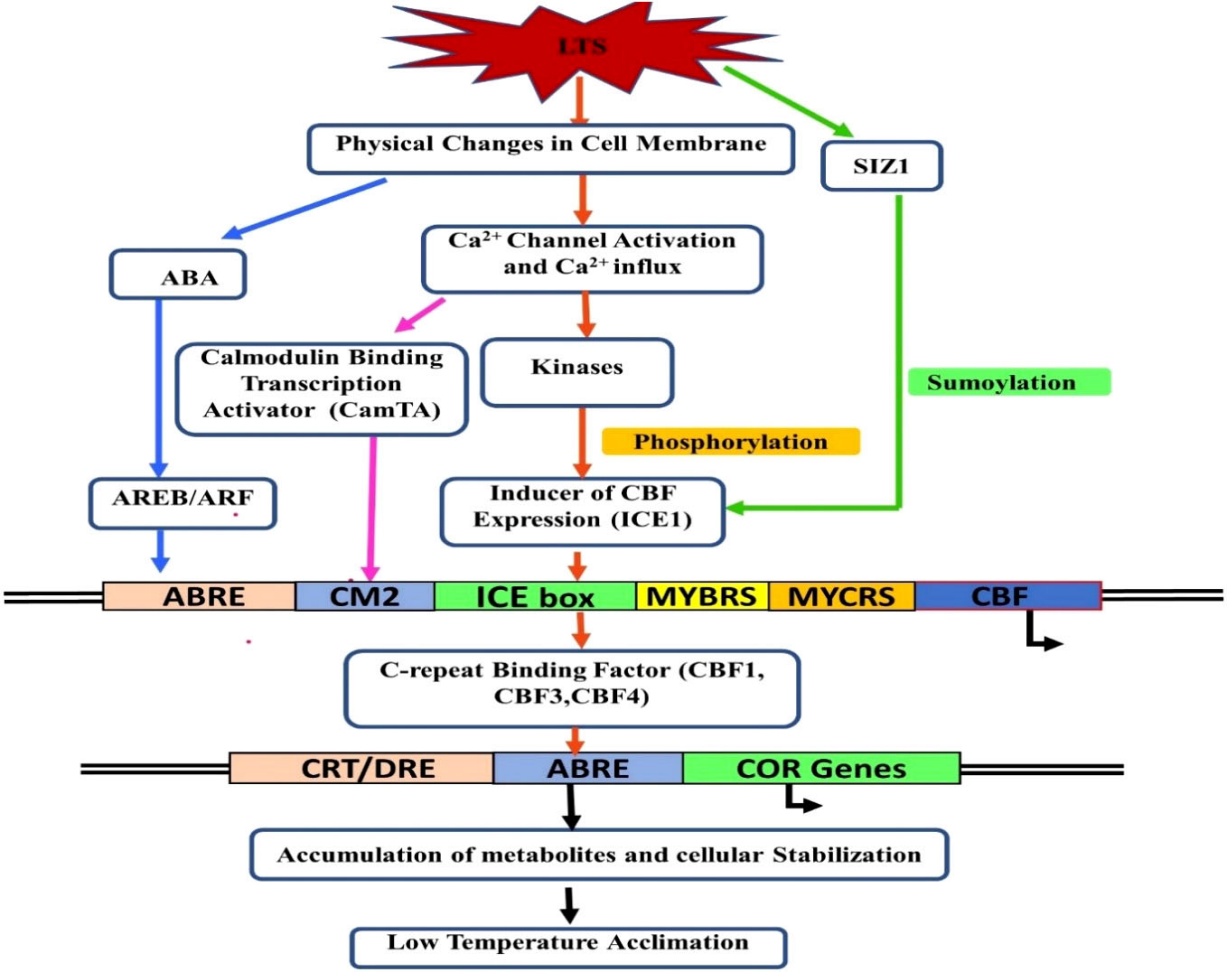
Signaling network for low-temperature stress acclimation in plants.

The response of a plant to LTS is to limit or eliminate the negative consequences of LT. It leads to biochemical and physiological changes in the plant cells, like membrane rigidification, lowering of enzyme kinetics, ROS generation, metabolic disequilibrium, etc. Plant species’ responses to cold stress vary, and the expression of several stress-associated genes are redirected to alter their metabolism accordingly (Chinnusamy et al., 2010; Guo et al., 2018). Plants can activate a cascade of processes that produce changes in gene expression (Zuther et al., 2019) and, as a result, promote biochemical and physiological adaptations that improve their resistance to low non-freezing temperatures. This process is known as cold acclimatization. Plants follow the adaptive mechanism against cold stress by changing the proteins at the translational level, the composition of the cell membrane, and triggering the ROS scavenging system. In addition, gene expression plays a crucial role in these adaptive mechanisms. The molecular mechanisms of cold stress tolerance and freezing tolerance have been studied in detail in *Arabidopsis* and winter cereals. It has been shown that temperate crops mainly acquire adaptive tolerance, whereas tropical and subtropical crops are vulnerable to cold stress (Chinnusamy et al., 2010). Many cold-regulated (*COR*) genes require specific signal transduction pathway activation for acclimatization under LTS (Thomashow, 1998) such as membrane linked receptor proteins, and the most extensively studied pathway: *ICE-CBF*-*COR* pathway (Zinta et al., 2022). (**Figure 3**).

The list of various gene families/gene(s) responsible for LTS tolerance in different fruit crops is listed in **Table 2**. The *COR* genes in *Arabidopsis* plants are regulated by CRT/DRE binding factors (*CBF*) at the transcription level to enhance the freezing tolerance (Jaglo-Ottosen et al., 1998; Gilmour et al., 2000). CBF related genes, namely, C*BF1*, *CBF2*, and *CBF3*, are rapidly and transiently expressed in response to LT (Gilmour et al., 1998). In the transgenic *Arabidopsis* plants, susceptible to cold stress, it has been found that ectopic synthesis of CBF proteins leads to constitutive expression of COR genes, resulting in enhanced freezing tolerance (Jaglo-Ottosen et al., 1998; Gilmour et al., 2000). The inducer of C-repeat binding factor (*CBF*) expression 1 (*ICE1*), part of the *CBF* transcriptional cascade, regulates transcription (Zhu et al., 2007). In addition, the activation of transcriptional cascades involving *CBF* is mediated by Ca^2+^ signaling (**Figure 3**). Under cold stress, the function of *ICE1* is to induce the constitutive expression of *CBF*s (Chinnusamy et al., 2007). PIF4 (Phytochrome Interacting Factor 4) and PIF7 (Phytochrome Interacting Factor 7) negatively regulate CBFs during the long day. On the other hand, when days are short, PIF4 and PIF7 are turned off, which makes plants more resistant to freezing. However, the actual mechanisms of photoperiod-induced repression of *CBF*s, *PIF4* and *PIF7*, are still not very evident; therefore, it may not be the sole mechanism engaged in acclimation processes (Fennell, 2014).

**Table 2:** Various gene (s) responsible for LTS tolerance in different fruit crops.

Sl. No.	Fruit crop	Gene (s)	Reference (s)
01	Grapevine (*Vitis vinifera*)	*VvCBF1, VvCBF2, VvCBF3, and VvCBF4*	Xiao et al. (2008)
02	Apple (*Malus x domestica*)	*MdCBF1, MdCBF2, MdCBF3, MdCBF4 and MdCBF5*	Feng et al. (2012)
03	Almond (*Prunus dulcis*)	*PdCBF1 and PdCBF2*	Barros et al. (2012)
04	Strawberry (*Fragaria × ananassa*)	*FaCBF1*	Owens et al. (2002)
05	Crab apple (*Malus baccata*)	*DREB1/CBF(Mb-DREB1)*	Yang et al. (2011)
06	Sour cherry (*Prunus cerasus*)	*PcCBF1*	Owens et al. (2002)
07	Sweet cherry (*Prunus avium*)	*PaCBF*	Kitashiba et al. (2004)
08	Blueberry (*Vaccinium corymbosum*)	*BB-CBF*	Polashock et al. (2010)
09	*Vitis riparia*	*VrCBF4 and VrCBF1a*	Xiao et al. (2008)
10	*Poncirus trifoliata*	*COR11, COR19*	Thomashow (2010)
11	Peach (*Prunus persica*)	*ppdhn1*	Artlip et al. (1997)
12	*Citrus unshiu*	*Cu-COR19*	Hara et al. (2004)
13	Banana *(Musa* spp.*)*	*MusaDHN-1*	Upendra et al. (2011)
14	Apple (*M. domestica*)	Dehydrin (*MdDHN*)	Liang et al. (2012)
15	Grape (*V. vinifera*)	Dehydrin (*DHN*)	Yang et al. (2012).
16	Banana *(Musa* spp.*)*	MaCslD4, MaCslA4/12, MaCslE2	Yuan et al. (2021)

**Figure 4:**
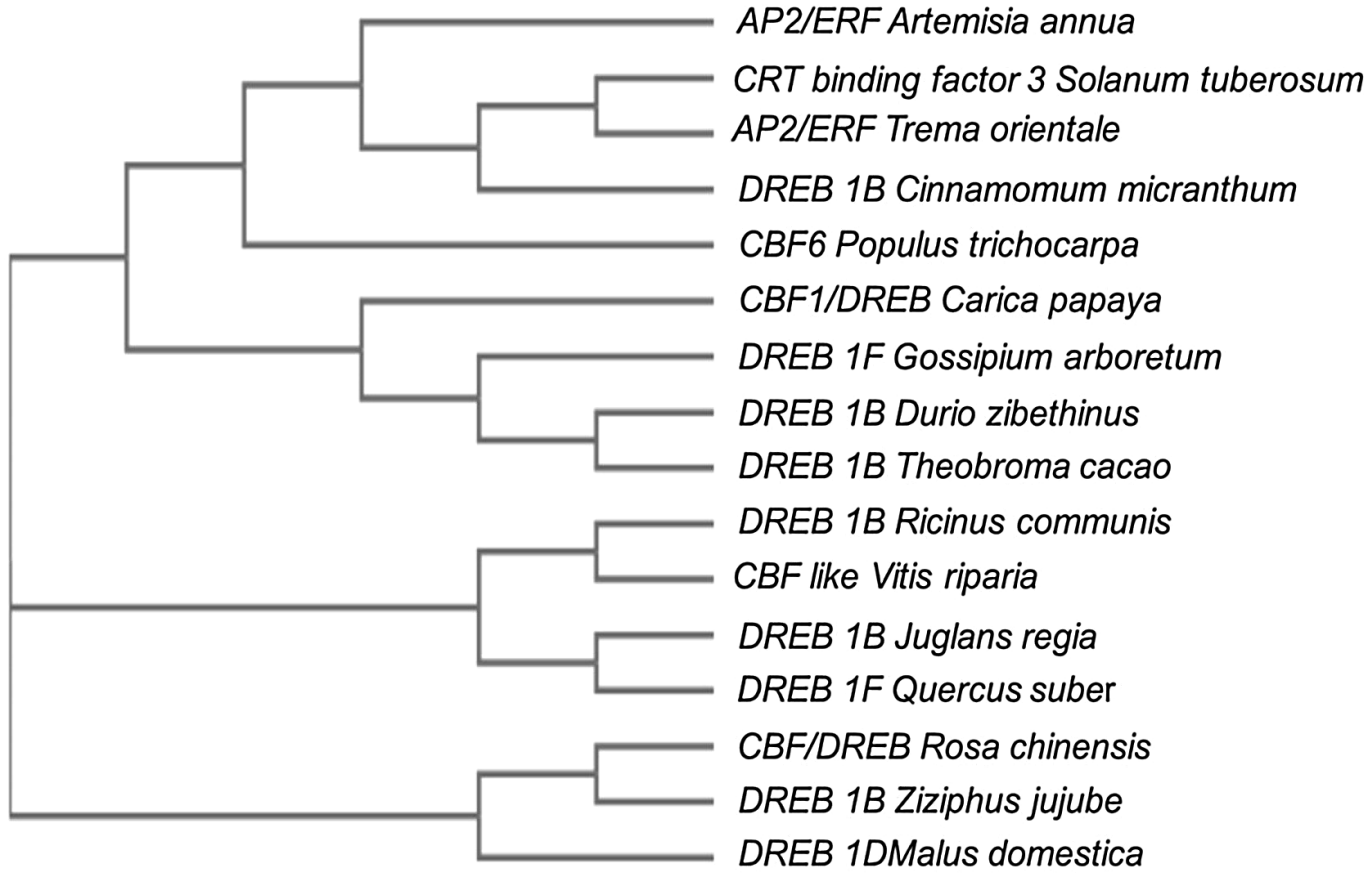
Phylogenetic tree of *CBFs* from different plants. *Carica papaya CBF1* (XP_021908755.1), *Juglans regia DREB 1B* Like (XP_018812676.1), *Quercus suber DREB 1 F* Like (XP_023913339.1), *Durio zibethinus DREB 1B* Like (XP_022744533.1), *Gossypium arboretum DREB 1F* Like (XP_017622119.1), *Ziziphus jujube DREB 1B* Like (XP_015901948.1), *Theobroma cacao DREB 1B* Like (EOY08142.1), *Cinnamomum micranthus DREB 1B* Like (RWR82367.1), *Ricinus communis DREB 1B* Like (EEF33807.1), *Populus trichocarpa* C-repeat binding factor 6 (ABO48367.1), *Vitis riparia CBF*-Like transcription factor (ARD27023.1), *Rosa chinensis* putative *CBF*/*DREB* transcription factor (ABQ53132.1), *Artemisia annua AP2*/*ERF* domain-containing protein (PWA50609), *Solanum tuberosum* CRT binding factor 3 (ACB45095.1), *Trema arientale AP2*/*ERF* domain-containing protein (PON57830.1), *Malus domestica DREB 1D* (XP_028948628.1) (FIGURE adapted from Maurya et al., 2020).

**Figure 5:**
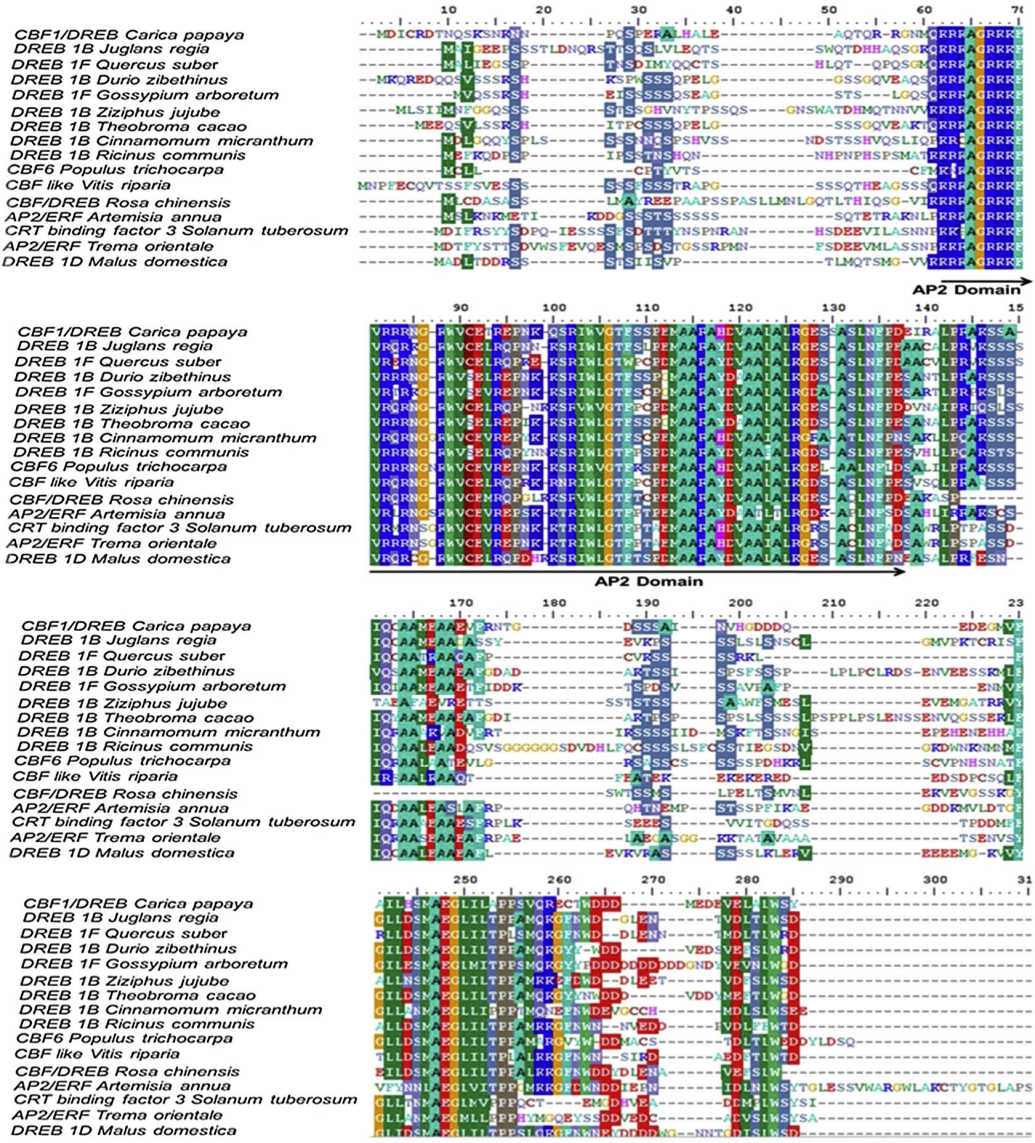
Alignment of the predicted amino acid sequences of the *Carica papaya CBF1*/*DREB* from with another plant *CBFs*/*DERB*. All the designated sequences showed conserved domain of AP2, the characteristics of c-repeat binding factors. (FIGURE adapted from Maurya et al., 2020).

Despite this, the *CBF*/*DREB1* dependent low-temperature signaling network is the most crucial regulatory system (Chinnusamy et al., 2007). Maurya et al. (2020) investigated the similarity of the putative amino acid sequence of *CBF1* of *Carica papaya* through a BLAST search. They reported higher similarities with *Quercus suber* (53%), *Malus domestica* (50%), *Durio zibethinus* (49%), *Theobroma cacao* (49%), *Ziziphus jujube* (48%) and *Rosa chinensis* (48%) (**Table 3**). Furthermore, the phylogenetic tree revealed the evolutionary link between the *CBFs* of different plants (**Figure 3**). The phylogenetic tree of *CBFs* was classified into three major groups, and *C. papaya CBF1*/*DREB* belonged to group-II, as presented in **Figure 4**. The comparison of amino acid sequence and multiple sequence alignment of *Carica papaya’s CBF1*/*DREB* protein and other plant species was shown in **Figure 5**. Dhekney et al. (2007) found cold-inducible regions and *CBF* sequences in *C. papaya* and *Vasconcellea* and reported sequence homology with *the Arabidopsis* genome. Recently, Maurya et al. (2020) studied the comparative expression profile of *CBF1* and *CBF2* gene transcripts under LTS and observed that the *CBF1* expression profiles was up-regulated while *CBF2* was down-regulated in six *C. papaya* genotypes (Red Lady, Pusa Nanha, P-7-2, P-7-9, P-7-14, and *V. cundinamarcensis*). In addition, they noted that *V. cundinamarcensis* had the highest expression profile of *CBF1*, followed by P-7-9, whereas the lowest level was found in Red Lady. On the other hand, the *CBF2* expression profile followed the opposite pattern. According to Zhu et al. (2007), high-temperature stress (HTS) had minimal influence on the *CpCBF2* expression profile, whereas LT inhibited it. The induction of *CBF1*/*DREB1B* and *CBF3*/*DREB1A* is simultaneous and before *CBF2*/*DREB1C* under cold stress (Medina et al., 2011). Fursova et al. (2009) found that overexpression of *ICE2* increased the expression profile of *CBF1/DREB1B* and improved freezing tolerance in *Arabidopsis.* Takuhara et al. (2011) found that the grapevine cold-induced transcription factors (*VvCBF2*, *VvCBF4*, *VvCBFL*, *VvZFPL*) also enhance LT tolerance in transgenic Arabidopsis plants. Transgenic strawberry leaves over expressing wheat acidic dehydrin gene (*Wcor410a*) improved their cold tolerance when exposed to 5^°^C (Houde et al., 2004). In addition, Hara et al. (2004) reported that a dehydrin gene, *i.e.,CuCOR19*, from *Citrus unshiu*, can operate as a hydroxyl radical scavenger under LT and found more tolerance to freezing when over expressed in *Nicotiana tabacum* (Hara et al., 2003). Apart from this, they also reported a negative association between the accumulation of *CuCOR19* and electrolyte ion leakage. The approximate size of peach’s dehydrin gene (*ppdhn1*) is 50 kDa, which encodes PCA60 protein, consisting of 472 amino acids. In the deciduous genotype, the protein accumulates earlier and is retained at higher levels for a longer time than in the evergreen genotype, indicating that it plays a role in cold tolerance (Artlip et al., 1997). Furthermore, the PCA60 protein acts as an anti-freeze protein that modifies ice crystal growth in the cell and is present in the cytosol and nucleus but absent in the vacuole when investigated under the light electron microscope (Wisniewski et al., 1999). LT tolerance is increased when MpRCI, a cold-tolerance-related plasma membrane protein gene of the ‘Dajiao banana cultivar, is overexpressed in transgenic tobacco. (Feng et al., 2009).

**Table 3:** Percent identity matrix of Carica papaya CBF1/DREB with other plants CBF/DREB factors (Table adapted from Maurya et al., 2020).

CBF1/DREB *Carica papaya*	100.00															
DREB 1B *Juglans regia*	47.55	100.00														
DREB 1F *Quercus suber*	53.16	59.67	100.00													
DREB 1F *Durio zibethinus*	49.48	59.69	55.00	100.00												
DREB 1F *Gossypium arboretum*	46.45	60.10	61.83	67.51	100.00											
DREB 1B *Ziziphus jujube*	48.80	59.02	61.58	66.50	82.27	100.00										
DREB 1B *Theobroma cacao*	49.50	47.06	53.63	50.00	45.59	43.78	100.00									
DREB 1B *Cinnamomum micranthum*	45.93	47.37	51.89	49.48	46.95	46.67	66.82	100.00								
DREB 1B *Ricinus communis*	45.58	51.44	54.79	52.82	48.61	48.84	52.68	51.42	100.00							
CBF6 *Populus trichocarpa*	47.09	51.00	52.91	51.05	53.02	53.70	44.72	43.27	50.00	100.00						
CBF like *Vitis riaparia*	45.60	51.91	58.60	51.18	56.52	54.89	43.24	45.79	50.54	53.59	100.00					
CBF/DREB *Rosa chinensis*	48.24	47.03	55.37	52.94	54.95	56.28	48.06	45.02	47.29	57.00	55.32	100.00				
A)2/ERF *Artemisia annua*	46.94	53.54	54.70	58.51	57.58	56.63	46.67	46.73	52.76	56.10	62.36	56.37	100.00			
CRT binding factor 3 *Solanum tuberosum*	46.63	55.88	54.59	52.36	56.87	56.87	44.66	45.50	49.53	53.49	62.23	53.85	62.44	100.00		
AP2/ERF *Trema orientale*	46.12	55.88	62.78	56.08	58.17	59.71	45.15	44.08	52.86	57.77	58.42	55.98	61.93	61.86	100.00	
DREB 1D *Malus domestica*	50.54	55.88	61.18	57.14	59.36	63.04	47.85	45.79	52.38	58.20	58.38	62.77	59.79	61.78	71.05	100.00

Overexpression of the *MbDREB1* transcription factor improved cold tolerance and other abiotic stresses in apples (Yang et al., 2011). Additionally, *Prunus persica CBF1* gene overexpression significantly enhanced freezing tolerance in transgenic apples (Wisniewski et al., 2011). Feng et al. (2012) studied the adaptive responses to cold tolerance in apples and discovered that the transcription factor MdCIbHLH1 plays an important role in the LTS.

Bananas are highly susceptible to chilling stress (Chen et al., 2020). Recently, Meng et al. (2020) reported that the accumulation of fasciclin-like arabinogalactan protein (*FLA*) in banana under low temperatures could improve chilling tolerance by facilitating cold signal pathways, which increases the biosynthesis of plant cell wall components. The results provided background information on *MaFLAs* and suggested their involvement in plant chilling tolerance and their potential as candidate genes to be targeted when breeding for cold tolerance. Yuan et al. (2021) reported that the *Csl* (*Cellulose synthase-like*) gene family imparts tolerance against LT in bananas by synthesizing a special kind of hemicellulose, an essential component of the cell wall, and strengthening the stiffness of the cell wall. The expression of genes *MaCslD4* (*Musa acuminate Cellulose synthase-like gene*), *MaCslA4*/*12*, and *MaCslE2* (member of *the Csl* gene family) were found to be significantly higher in the tolerant variety than in the susceptible banana variety under LTS (Yuan et al., 2021).

The commercially important diversity of citrus is available for cultivation. However, it has been observed that most cold-acclimated citrus cultivars can only sustain temperature variations of -4° to -10°C. However, *Poncirus trifoliata*, a citrus relative, is a genetic resource for cold-hardiness that can survive at temperatures as low as -26°C (Yelenosky, 1985). Zhang et al. (2005) studied the gene expression profile of *P. trifoliata* by mRNA DDRT-PCR (differential display-reverse transcription) and quantitative relative-RT-PCR in a cold acclimation temperature regime. The up-regulated genes showed high similarities at the amino acid level with genes with known functions like water channel protein, betaine or proline transporter, early light-induced protein, nitrate transporter, aldo-ketoreductase, tetratricopeptide-repeat protein, F-box protein, ribosomal (r) protein L15. These cold-acclimation up-regulated genes of *P. trifoliata* are also regulated by osmotic and photo-oxidative signals in other plants.

Anthocyanins, a water-soluble pigment available across the plant kingdom, have a significant role in mitigating cold stress by increasing their content in the cell and maintaining the osmotic balance to improve the chilling tolerance (Chalker-Scott, 1999; Hao et al., 2009). Lo Piero et al. (2005) reported an accumulation of anthocyanin in red-orange juice vesicles under LTS. The biosynthesis pathway of anthocyanin is complex and involves several structural genes: *PAL* (phenylalanine ammonia-lyase), *CHS* (chalcone synthase), *DFR* (dihydro flavonol 4-reductase), and *UFGT* (UDP-glucose flavonoid glucosyltransferase), which are strongly triggered during LTS.

According to Crifo et al. (2011), cold stress causes transcriptome changes in blood oranges that trigger enhancement of flavonoid biosynthesis, including the metabolic pathways of anthocyanin biosynthesis. Similarly, in red-skinned grapes, cold stress promoted anthocyanin biosynthesis but did not increase the accumulation of endogenous levels of abscisic acid (Gao-Takai et al., 2019). Using an RNA-seq study of leaves from the ‘Gala’ apple variety after exposure to LT (16°C), Song et al. (2019) identified additional regulatory genes ROS1 in apple (*M. domestica*) that may be involved in controlling LT-induced anthocyanin production.

## Conclusion

LTS unfavorably affects the fruit crop’s overall growth and development because it affects almost all aspects of cellular activity and, ultimately the quality production of the crop. However, according to the current review, the method of comprehending low-temperature damage have been adequately tried by researchers on many field crops, while very limited studies have been conducted on fruit crops. These include primarily temperate fruit crops and some tropical crops like bananas. Therefore, new avenues like the role and use of aquaporin genes to alleviate the LTS need to be explored in tropical and sub-tropical fruit crops. Furthermore, under open and simulated low-temperature conditions, more detailed research is required to elucidate the underlying physio-biochemical and molecular mechanisms for LTS tolerance and resistance in fruit crops. These strategies will immensely help in genetic improvement and standardizing cultural practices for successful cultivation of the fruit crops, particularly in the erratic weather conditions of sub-tropical and tropical regions.

## Author contributions

AKG, NKM, SG, and KB conceived the idea of the review and prepared the initial outline, and wrote the first draft. AKG and NKM had major and equal contributions to the overall preparation of the manuscript. SKS, JP, SP, AKG, VC, PK, SP, DB, and CK gathered the literature and contributed to writing the different sections. SKS, SG, KB, and RMS provided technical guidance and editing support. All authors have read and approved the manuscript.

## References

[B1] Achard P. Cheng H. De Grauwe L. Decat J. Schoutteten H. Moritz T. Harberd N. P. (2006). Integration of plant responses to environmentally activated phytohormonal signals. Science 311(5757), 91–94. 16400150 10.1126/science.1118642

[B2] Achard P. Gong F. Cheminant S. Alioua M. Hedden P. Genschik P. (2016). The cold-inducible CBF1 factor–dependent signaling pathway modulates the accumulation of the growth-repressing DELLA proteins *via* its effect on gibberellin metabolism. Plant Cell 20, 2117–2129. doi: 10.1105/tpc.108.058941 PMC2553604 18757556

[B3] Ahmad P. Jaleel C. A. Salem M. A. Nabi G. Sharma S. (2010). Roles of enzymatic and nonenzymatic antioxidants in plants during abiotic stress. Crit. Rev. Biotechnol. 30 (3), 161–175. doi: 10.3109/07388550903524243 20214435

[B4] Allen D. J. Ort D. R. (2001). Impacts of chilling temperatures on photosynthesis in warm-climate plants. Trends Plant Sci. 6, 36–42. doi: 10.1016/S1360-1385(00)01808-2 11164376

[B5] Alonso A. Queiroz C. S. Magalhaes A. C. (1997). Chilling stress leads to increased cell membrane rigidity in roots of coffee (Coffea arabica l.) seedlings. Biochim. Biophys. Acta 1323, 75–84. doi: 10.1016/S0005-2736(96)00177-0 9030214

[B6] Aroca R. Irigoyen J. J. Sanchez-Diaz M. (2001). Photosynthetic characteristics and protective mechanisms against oxidative stress during chilling and subsequent recovery in two maize varieties differing in chilling sensitivity. Plant Sci. 161, 719–726. doi: 10.1016/S0168-9452(01)00460-5

[B7] Arora R. (2018). Mechanism of freeze-thaw injury and recovery: a cool retrospective and warming up to new ideas. Plant Sci. 270, 301–313. doi: 10.1016/j.plantsci.2018.03.002 29576084

[B8] Artlip T. S. Callahan A. M. Bassett C. L. Wisniewski M. E. (1997). Seasonal expression of a dehydrin gene in sibling deciduous and evergreen genotypes of peach (Prunus persica [L.] batsch). Plant Mol. Biol. 33, 61–70. doi: 10.1023/A:1005787909506 9037159

[B9] Awada M. (1958). “Relationships of minimum temperature and growth rate with sex expression of papaya plants (Carica papaya l.),” in Hawaii Agricultural experiment station technical bulletin, vol. 38. (Honolulu: University of Hawaii).

[B10] Baiges I. Schaffner A. R. Affenzeller M. J. Mas A. (2002). Plant aquaporins. Physiol. Plant 115, 175–182. doi: 10.1034/j.1399-3054.2002.1150201.x 12060233

[B11] Barlow K. K. Lee J. W. Vesk M. RL B. AR F. MM C. (1974). “Mechanisms of regulation of plant growth,” in The royal society of new Zealand, Bieleski R. L. Ferguson A. R. Cresswell M. M. Eds. (Wellington: Royal Society of New Zealand) 793–797.

[B12] Barros R. S. da Se Mota J. W. Da Matta F. M. Maestri M. (1997). Decline of vegetative growth in coffea arabica l. @ in relation to leaf temperature, water potential and stomatal conductance. Field Crops Res. 54, 65–72. doi: 10.1016/S0378-4290(97)00045-2

[B13] Barros P. M. Gonçalves N. Saibo N. J. Oliveira M. M. (2012). Cold acclimation and floral development in almond bud break: insights into the regulatory pathways. J. Exp. Bot. 63, 4585–4596. doi: 10.1093/xb/ers144 22685307

[B14] Bauer H. Wierer R. Hatheway W. H. Larcher W. (1985). Photosynthesis of coffea arabica after chilling. Physiol. Plant 64, 449–454. doi: 10.1111/j.1399-3054.1985.tb08521.x

[B15] Beck E. H. Fettig S. Knake C. Hartig K. Bhattarai T. (2007). Specific and unspecific responses of plants to cold and drought stress. J. Biosci. 32, 501–510. doi: 10.1007/s12038-007-0049-5 17536169

[B16] Bies-Etheve N. Gaubier-Comella P. Debures A. Lasserre E. Jobet E. Raynal M. . (2008). Inventory, evolution and expression profiling diversity of the LEA (late embryogenesis abundant) protein gene family in arabidopsis thaliana. Plant Mol. Biol. 67, 107–124. doi: 10.1007/s11103-008-9304-x 18265943

[B17] Bowen P. Shellie K. C. Mills L. Willwerth J. Bogdanoff C. Keller M. (2016). Abscisic acid form, concentration, and application timing influence phenology and bud cold hardiness in merlot grapevines. Can. J. Plant Sci. 96, 347–359. doi: 10.1139/cips-2015-0257

[B18] Campos P. S. Quartin V. Ramalho J. C. Nunes M. ,. A. (2003). Electrolyte leakage and lipid degradation account for cold sensitivity in leaves of coffea sp. plants. J. Plant Physiol. 160, 283–292. doi: 10.1078/0176-1617-00833 12749085

[B19] Cao S. Zheng Y. Wang K. Jin P. Rui H. (2009). Methyl jasmonate reduces chilling injury and enhances antioxidant enzyme activity in postharvest loquat fruit. Food Chem. 115, 1458–1463. doi: 10.1016/j.foodchem.2009.01.082

[B20] Chalker-Scott L. (1999). Environmental significance of anthocyanins in plant stress responses. Photochem. Photobiol. 70, 1–9. doi: 10.1111/j.1751-1097.1999.tb01944.x

[B21] Chen Y. Z. Patterson B. D. (1985). Ethylene and 1-aminocyclopropane-1-carboxylic acid as indicators of chilling sensitivity in various plant species. Funct. Plant Biol. 12, 377–385. doi: 10.1071/PP9850377

[B22] Chen L. Zhao X. Wu J. E. He Y. Yang H. (2020). Metabolic analysis of salicylic acid-induced chilling tolerance of banana using NMR. Food Res. Int. 128, 108796. doi: 10.1016/j.foodres.2019.108796 31955759

[B23] Chinnusamy V. Zhu J. K. Sunkar R. (2010). Gene regulation during cold stress acclimation in plants. Meth. Mol. Biol. 639, 39–55. doi: 10.1007/978-1-60761-702-0_3 PMC3064467 20387039

[B24] Chinnusamy V. Zhu J. Zhu J. K. (2007). Cold stress regulation of gene expression in plants. Trends Plant Sci. 12, 444–451. doi: 10.1016/j.tplants.2007.07.002 17855156

[B25] Criddle R. S. Breidenbach R. W. Lewis E. A. Eatough D. J. Hansen L. D. (1988). Effects of temperature and oxygen depletion on metabolic rates of tomato and carrot cell cultures and cuttings measured by calorimetry. Plant Cell Environ. 11, 695–701. doi: 10.1111/j.1365-3040.1988.tb01152.x

[B26] Crifo T. Puglisi I. Petrone G. Recupero G. R. Piero A. R. L. (2011). Expression analysis in response to low temperature stress in blood oranges, implication of the flavonoid biosynthetic pathway. Gene 476, 1–9. doi: 10.1016/j.gene.2011.02.005 21349317

[B27] Davanport T. L. (2009). “Reproductive physiology,” in The mango, 2nd edn. botany, production and uses. Ed. Litz R. E. (London: CABI).

[B28] Dhekney S. A. Litz R. E. Moraga D. Yadav A. (2007). Is it possible to induce cold tolerance in papaya through genetic transformation? Acta Hortic., vol. 738, 159–164. doi: 10.17660/ActaHortic.2007.738.13

[B29] Ding F. Wang R. (2018). Amelioration of postharvest chilling stress by trehalose in pepper. Scientia Hortic. 232, 52–56. doi: 10.1016/j.scienta.2017.12.053

[B30] Ding Y. Yang S. (2022). Surviving and thriving: How plants perceive and respond to temperature stress. Dev. Cell 57, 947–958. doi: 10.1016/j.devcel.2022.03.010 35417676

[B31] Donnamaria M. C. Howard E. I. Grigera J. R. (1994). Interaction of water with α, α-trehalose in solution, molecular dynamics simulation approach. J. Chem. Soc. Faraday Trans. 90, 2731–2735. doi: 10.1039/FT9949002731

[B32] Dorffling K. Schulenburg S. Lesselich G. Dorffling H. (1990). Abscisic acid and proline levels in cold hardened winter wheat leaves in relation to variety-specific differences in freezing resistance. J. Agron. Crop Sci. 165, 230–239. doi: 10.1111/j.1439-037x.1990.tb00857.x

[B33] Ehlert B. Hincha D. K. (2008). Chlorophyll fluorescence imaging accurately quantifies freezing damage and cold acclimation responses in arabidopsis leaves. Plant Meth. 4, 1–7. doi: 10.1186/1746-4811-4-12 PMC2430023 18505561

[B34] Elbein A. D. Pan Y. T. Pastuszak I. Carroll D. (2003). New insights on trehalose: a multifunctional molecule. Glycob. 13, 17–27. doi: 10.1093/glycob/cwg047 12626396

[B35] Fariduddin Q. Yusuf M. Chalkoo S. Hayat S. Ahmad A. (2011). 28-homobrassinolide improves growth and photosynthesis in cucumis sativus l. through an enhanced antioxidant system in the presence of chilling stress. Photosynthetica 49, 55–64. doi: 10.1007/s11099-011-0022-2

[B36] Fedotova M. V. (2019). Compatible osmolytes-bioprotectants: is there a common link between their hydration and their protective action under abiotic stresses? J. Mol. Liq. 292, 111339. doi: 10.1016/j.molliq.2019.1111339

[B37] Feng D. R. Liu B. Li W. Y. He Y. M. Qi K. B. Wang H. B. . (2009). Over-expression of a cold-induced plasma membrane protein gene (MpRCI) from plantain enhances low temperature-resistance in transgenic tobacco. Environ. Exp. Bot. 65, 395–402. doi: 10.1016/j.envexpbot.2008.12.009

[B38] Feng X. M. Zhao Q. Zhao L. L. Qiao Y. Xie X. B. Li H. F. . (2012). The cold-induced basic helix-loop-helix transcription factor gene MdCIbHLH1 encodes an ICE-like protein in apple. BMC Plant Biol. 12, 1–14. doi: 10.1186/1471-2229-12-22 22336381 PMC3352023

[B39] Fennell A. (2014). Genomics and functional genomics of winter low temperature tolerance in temperate fruit crops. Crit. Rev. Plant Sci. 33, 125–140. doi: 10.1080/07352689.2014.870410

[B40] Fernandez O. Theocharis A. Bordiec S. Feil R. Jacquens L. Clement C. . (2012). Burkholderia phytofirmans PsJN acclimates grapevine to cold by modulating carbohydrate metabolism. Mol. Plant Microbe Interact. 25, 496–504. doi: 10.1094/MPMI-09-11-0245 22409157

[B41] Foyer C. H. Noctor G. (2011). Ascorbate and glutathione: the heart of the redox hub. Plant Physiol. 155 (1), 2–18. doi: 10.1104/pp.110.167569 21205630 PMC3075780

[B42] Fursova O. V. Pogorelko G. V. Tarasov V. A. (2009). Identification of ICE2, a gene involved in cold acclimation which determines freezing tolerance in arabidopsis thaliana. Gene 429, 98–103. doi: 10.1016/j.gene.2008.10.016 19026725

[B43] Gao-Takai M. Katayama-Ikegami A. Matsuda K. Shindo H. Uemae S. Oyaizu M. (2019). A low temperature promotes anthocyanin biosynthesis but does not accelerate endogenous abscisic acid accumulation in red-skinned grapes. Plant Sci. 283, 165–176. doi: 10.1016/j.plantsci.2019.01.015 31128686

[B44] Geng J. Wei T. Wang Y. Huang X. Liu J. H. (2019). Overexpression of PtrbHLH, a basic helix-loop-helix transcription factor from poncirus trifoliata, confers enhanced cold tolerance in pummelo (Citrus grandis) by modulation of H2O2 level *via* regulating a CAT gene. Tree Physiol. 39, 2045–2054. doi: 10.1093/treephys/tpz081 31330032

[B45] Gilmour S. J. Zarka D. G. Stockinger E. J. Salazar M. P. Houghton J. M. Thomashow M. F. (1998). Low temperature regulation of the Arabidopsis CBF family of AP2 transcriptional activators as an early step in cold - induced COR gene expression. Plant Journal 16, 433–442. doi: 10.1046/j.1365-313x.1998.00310.x 9881163

[B46] Gilmour S. J. Sebolt A. M. Salazar M. P. Everard J. D. Thomashow M. F. (2000). Overexpression of the arabidopsis CBF3 transcriptional activator mimics multiple biochemical changes associated with cold acclimation. Plant Physiol. 124, 1854–1865. doi: 10.1104/pp.124.4.1854 11115899 PMC59880

[B47] Glaszmann J. C. Kaw R. N. Khush G. S. (1990). Genetic divergence among cold tolerant rices (Oryza sativa l.). Euphytica 45, 95–104. doi: 10.1007/BF00033276

[B48] Goddijn O. J. Van Dun K. (1999). Trehalose metabolism in plants. Trends Plant Sci. 4, 315–319. doi: 10.1016/S1360-1385(99)01446-6 10431221

[B49] Gonzalez-Aguilar G. A. Buta J. G. Wang C. Y. (2001). Methyl jasmonate reduces chilling injury symptoms and enhances colour development of ‘Kent’ mangoes. J. Sci. Food Agric. 81, 1244–1249. doi: 10.1002/jsfa.933

[B50] Gonzalez-Aguilar G. A. Fortiz J. Cruz R. Baez R. Wang C. Y. (2000). Methyl jasmonate reduces chilling injury and maintains postharvest quality of mango fruit. J. Agric. Food Chem. 48, 515–519. doi: 10.1021/jf9902806 10691668

[B51] Gonzalez-Aguilar G. A. Tiznado-Hernandez M. E. Zavaleta-Gatica R. Martınez-Tellez M. A. (2004). Methyl jasmonate treatments reduce chilling injury and activate the defense response of guava fruits. Biochem. Biophys. Res. Commun. 313, 694–701. doi: 10.1016/j.bbrc.2003.11.165 14697246

[B52] Grau A. Halloy S. (1997). Effect of chilling on CO2 gas-exchange in carica papaya l. and carica quercifolia (A. st. hil.) solms. J. Plant Physiol. 150, 475–480. doi: 10.1016/S0176-1617(97)80101-0

[B53] Grewal J. S. Singh S. N. (1980). Effect of potassium nutrition on frost damage and yield of potato plants on alluvial soils of the punjab (India). Plant Soil 57, 105–110. doi: 10.1007/Bf02139646

[B54] Guo X. Liu D. Chong K. (2018). Cold signaling in plants: Insights into mechanisms and regulation. J. Integr. Plant Biol. 60 (9), 745–756. doi: 10.1111/jipb.12706 30094919

[B55] Guy C. L. Niemi K. J. Brambl R. (1985). Altered gene expression during cold acclimation of spinach. Proc. Natl. Acad. Sci. 82, 3673–3677. doi: 10.1073/pnas.82.11.3673 3858842 PMC397849

[B56] Hakerlerler H. Oktay M. Eryuce N. Yagmur B. (1997). “Effect of potassium sources on the chilling tolerance of some vegetable seedlings grown in hotbeds,” in Food security in the WANA region, the essential need for balanced fertilization. Ed. Johnston A. E. (Switzerland: International Potash Institute), 317–327.

[B57] Hamdani S. Yaakoubi H. Carpentier R. (2011). Polyamines interaction with thylakoid proteins during stress. J. Photochem. Photobiol. 104, 314–319. doi: 10.1016/j.jphotobiol.2011.02.007 21377374

[B58] Hamman R. A. Dami I. E. Walsh T. M. Stushnoff C. (1996). Seasonal carbohydrate changes and cold hardiness of Chardonnay and Riesling grapevines. Am. J. Enol. Vitic. 47, 31–36.

[B59] Hao W. Arora R. Yadav A. K. Joshee N. (2009). Freezing tolerance and cold acclimation in guava (Psidium guajava l.). Hort Sci. 44, 1258–1266. doi: 10.21273/HORTSCI.44.5.1258

[B60] Haq N. (2006). “Jackfruit (Artocarpus heterophyllus),” in Tropical fruit trees, Southampton centre for underutilised crops. Eds. Williams J. T. Smith R. W. Dunsiger Z. (Southampton, UK: University of Southampton).

[B61] Hara M. Fujinaga M. Kuboi T. (2004). Radical scavenging activity and oxidative modification of citrus dehydrin. Plant Physiol. Biochem. 42, 657–662. doi: 10.1016/j.plaphy.2004.06.004 15331095

[B62] Hara M. Terashima S. Fukaya T. Kuboi T. (2003). Enhancement of cold tolerance and inhibition of lipid peroxidation by citrus dehydrin in transgenic tobacco. Planta 217, 290–298. doi: 10.1007/s00425-003-0986-7 12783337

[B63] Harrington J. F. Kihara G. M. (1960). Chilling injury of germinating muskmelon and pepper seed. Proc. Am. Soc Hortic. Sci. 75, 485–489.

[B64] Hasanuzzaman M. Nahar K. Fujita M. (2013). “Extreme temperatures, oxidative stress and antioxidant defense in plants,” in Abiotic stress-plant responses and applications in agriculture, vol. 13 . Eds. Vahdati K. Leslie C. (Rijeka: InTech), 169–205.

[B65] Hayat S. Hayat Q. Alyemeni M. N. Wani A. S. Pichtel J. Ahmad A. (2012). Role of proline under changing environments: a review. Plant Signal. Behav. 7, 1456–1466. doi: 10.4161/psb.21949 22951402 PMC3548871

[B66] He W. D. Gao J. Dou T. X. Shao X. H. Bi F. C. Sheng O. . (2018). Early cold-induced peroxidases and aquaporins are associated with high cold tolerance in dajiao (Musa spp. ‘Dajiao’). Front. Plant Sci. 9. doi: 10.3389/fpls.2018.00282 PMC5852111 29568304

[B67] Herner R. C. (1990). Chilling injury of horticultural crops. Ed. Wang C. V. (Boca Raton, FL., USA: CRC Press), 51–69.

[B68] Houde M. Dallaire S. N'Dong D. Sarhan F. (2004). Overexpression of the acidic dehydrins WCOR410 improves freezing tolerance in transgenic strawberry leaves. Plant Biotechnol. J. 2, 381–387. doi: 10.1111/j.1467-7652.2004.00082.x 17168885

[B69] Hsu S. Y. Hsu Y. T. Kao C. H. (2003). The effect of polyethylene glycol on proline accumulation in rice leaves. Biol. Plant 46, 73–78. doi: 10.1023/A:1022362117395

[B70] Jaglo-Ottosen K. R. Gilmour S. J. Zarka D. G. Schabenberger O. Thomashow M. F. (1998). Arabidopsis CBF1 overexpression induces COR genes and enhances freezing tolerance. Science 280, 104–106. doi: 10.1126/science.280.5360.104 9525853

[B71] Jan N. Andrabi K. I. (2009). Cold resistance in plants: A mystery unresolved. Electronic J. Biotechnol. 12, 14–15. doi: 10.4067/S0717-34582009000300014

[B72] Janska A. Mars P. Zelenkova S. Ovesna J. (2009). Cold stress and acclimation-what is important for metabolic adjustment? Plant Biol. 12, 395–405. doi: 10.1111/j.1438-8677.2009.00299.x 20522175

[B73] Jogawat A. (2019). “Osmolytes and their role in abiotic stress tolerance in plants,” in Molecular plant abiotic stress: Biology and biotechnology. Eds. Roychoudhury A. Tripathi D. (Hoboken, NJ, USA: John Wiley and Sons Ltd), 91–104. doi: 10.1002/9781119463665.ch5

[B74] Joubert A. S. Thomas D. D. S. (1965). The cashew nut. Farming south Africa 40, 6–7.

[B75] Kafkafi U. (1990). “Impact of potassium in relieving plants from climatic and soil-induced stresses,” in Food security in the WANA region, the essential need for balanced fertilization. Ed. Johnston A. E. (Basel: International Potash Institute), 317–327.

[B76] Kaplan F. Kopka J. Sung D. Y. Zhao W. Popp M. Porat R. . (2007). Transcript and metabolite profiling during cold acclimation of arabidopsis reveals an intricate relationship of cold-regulated gene expression with modifications in metabolite content. Plant J. 50, 967–981. doi: 10.1111/j.1365-313X.2007.03100.x 17461790

[B77] Kempf B. Bremer E. (1998). Stress responses of bacillus subtilis to high osmolarity environments, uptake and synthesis of osmoprotectants. J. Biosci. 23, 447–455. doi: 10.1007/BF02936138

[B78] Kerchev P. I. Van Breusegem F. (2022). Improving oxidative stress resilience in plants. Plant J. 109 (2), 359–372. doi: 10.1111/tpj.15493 34519111

[B79] Kishor P. K. Sangam S. Amrutha R. N. Laxmi P. S. Naidu K. R. Rao K. S. . (2005). Regulation of proline biosynthesis, degradation, uptake and transport in higher plants, its implications in plant growth and abiotic stress tolerance. Curr. Sci. 88, 424–438.

[B80] Kitashiba H. Ishizaka T. Isuzugawa K. Nishimura K. Suzuki T. (2004). Expression of a sweet cherry DREB1/CBF ortholog in arabidopsis confers salt and freezing tolerance. J. Plant Physiol. 161, 1171–1176. doi: 10.1016/j.jplph.2004.04.008 15535126

[B81] Kotzabasis K. Christakishampsas M. D. Roubelakisangelakis K. A. (1993). A narrow-bore HPLC method for the identification and quantitation of free, conjugated, and bound polyamines. Anal. Biochem. 214, 484–489. doi: 10.1006/abio.1993.1526 8109737

[B82] Kumar V. Yadav S. K. (2009). Proline and betaine provide protection to antioxidant and methylglyoxal detoxification systems during cold stress in camellia sinensis (L.) o. kuntze. Acta Physiol. Plant 31, 261–269. doi: 10.1007/s11738-008-0227-6

[B83] Kushad M. M. Yelenosky G. (1987). Evaluation of polyamine and proline levels during low temperature acclimation of citrus. Plant Physiol. 84, 692–695. doi: 10.1104/pp.84.3.692 16665504 PMC1056652

[B84] Lado J. Rodrigo M. J. López-Climent M. Gómez-Cadenas A. Zacarías L. (2016). Implication of the antioxidant system in chilling injury tolerance in the red peel of grapefruit. Postharvest Biol. Technol. 111, 214–223. doi: 10.1016/j.postharvbio.2015.09.01

[B85] Lee S. H. Chung G. C. Jang J. Y. Ahn S. J. Zwiazek J. J. (2012). Overexpression of PIP2; 5 aquaporin alleviates effects of low root temperature on cell hydraulic conductivity and growth in arabidopsis. Plant Physiol. 159, 479–488. doi: 10.1104/pp.112.194506 22434042 PMC3375980

[B86] Lee D. H. Lee C. B. (2000). Chilling stress-induced changes of antioxidant enzymes in the leaves of cucumber, in gel enzyme activity assays. Plant Sci. 159, 75–85. doi: 10.1016/S0168-9452(00)00326-5 11011095

[B87] Levitt J. (1980). Responses of plant to environmental stress chilling, freezing, and high temperature stresses, second ed Vol. 1 (New York: Academic Press), 23–64.

[B88] Li J. Ban L. Wen H. Wang Z. Dzyubenko N. Chapurin V. . (2015). An aquaporin protein is associated with drought stress tolerance. Biochem. Biophys. Res. Commun. 459, 208–213. doi: 10.1016/j.bbrc.2015.02.052 25701792

[B89] Li S. Dami I. E. (2016). Responses of vitis vinifera ‘Pinot gris’ grapevines to exogenous abscisic acid (aba), i. yield, fruit quality, dormancy, and freezing tolerance. J. Plant Growth Reg. 35, 245–255. doi: 10.1007/s00344-015-9529-2

[B90] Liang D. Xia H. Wu S. Ma F. (2011). Genome-wide identification and expression profiling of dehydrin gene family in Malus domestica. Mol. Biol. Rep. 59, 10759–10768. doi: 10.1007/s11033-012-1968-2 23053973

[B91] Lin H. Liao Z. Zhang L. Yu Q. (2016). Transcriptome analysis of the male-to-hermaphrodite sex reversal induced by low temperature in papaya. Tree Genet. Genomes 12, 94. doi: 10.1007/s11295-016-1055-2

[B92] Livingston D. P. Hincha D. K. Heyer A. G. (2009). Fructan and its relationship to abiotic stress tolerance in plants. Cell. Mol. Life Sci. 66, 2007–2023. doi: 10.1007/s00018-009-0002-x 19290476 PMC2705711

[B93] Lopez-Zaplana A. Martinez-Garcia N. Carvajal M. Barzana G. (2022). Relationships between aquaporins gene expression and nutrient concentrations in melon plants (Cucumis melo l.) during typical abiotic stresses. Environ. Exp. Bot. 195, 104759. doi: 10.1016/j.envexpbot.2021.104759

[B94] Lo Piero A. R. Puglisi I. Rapisarda P. Petrone G. (2005). Anthocyanins accumulation and related gene expression in red orange fruit induced by low temperature storage. J. Agric. Food Chem. 53, 9083–9088. doi: 10.1021/jf051609s 16277406

[B95] Louis H. Annappan R. S. (1980). Environmental effects on yield of coconut palm. Indian Coconut J. 12, 1–4.

[B96] Luo Z. Chen C. Xie J. (2011). Effect of salicylic acid treatment on alleviating postharvest chilling injury of ‘Qingnai’plum fruit. Postharvest Biol. Technol. 62, 115–120. doi: 10.1016/j.postharvbio.2011.05.012

[B97] Maestri M. Barros R. S. (1977). “Coffee,” in Ecophysiology of tropical crops. Eds. de Alvim P. T. Kozlowski T. T. (New York: Academic Press), 249–278.

[B98] Mahajan S. Tuteja N. (2005). Cold, salinity and drought stresses: An overview. Arch. Biochem. Biophys. 444, 139–158. doi: 10.1016/j.abb.2005.10.018 16309626

[B99] Martin L. J. Douglas G. (1979). “Proceedings of an international seminar on low temperature stress in crop plants held at the East-West center,” in Low temperature stress in crop plants: the role of the membrane (Honolulu, Hawaii: Academic Press rapid manuscript reproduction (USA).

[B100] Matteucci M. D'angeli S. Errico S. Lamanna R. Perrotta G. Altamura M. M. (2011). Cold affects the transcription of fatty acid desaturases and oil quality in the fruit of olea europaea l. genotypes with different cold hardiness. J. Exp. Bot. 62, 3403–3420. doi: 10.1093/jxb/err013 21357772 PMC3130166

[B101] Maurel C. Boursiac Y. Luu D. T. Santoni V. Shahzad Z. Verdoucq L. (2015). Aquaporins in plants. Physiol. Rev. 95, 1321–1358. doi: 10.1152/physrev.00008.2015 26336033

[B102] Maurya N. K. Goswami A. K. Singh S. K. Prakash J. Goswami S. Chinnusamy V. . (2020). Studies on expression of CBF1 and CBF2 genes and antioxidant enzyme activities in papaya genotypes exposed to low temperature stress. Sci. Hortic. 261, 108914. doi: 10.1016/j.scienta.2019.108914

[B103] Maurya N. K. Goswami A. K. Singh S. K. Prakash J. Goswami S. Chinnusamy V. . (2020). Low temperature stress induced changes in the seedling growth and nutrient content of papaya genotypes. Indian J. Hortic. 77, 80–87. doi: 10.5958/0974-0112.2020.00006.7

[B104] Maxwell K. Johnson G. N. (2000). Chlorophyll fluorescence- a practical guide. J. Exp. Bot. 51, 659–668. doi: 10.1093/jexbot/51.345.659 10938857

[B105] Medina J. Catala R. Salinas J. (2011). The CBFs: three arabidopsis transcription factors to cold acclimate. Plant Sci. 180, 3–11. doi: 10.1016/j.plantsci.2010.06.019 21421341

[B106] Meng J. Hu B. Yi G. Li X. Chen H. Wang Y. . (2020). Genome-wide analyses of banana fasciclin-like AGP genes and their differential expression under low-temperature stress in chilling sensitive and tolerant cultivars. Plant Cell Rep. 39, 693–708. doi: 10.1007/s00299-020-02524-0 32128627

[B107] Mirdehghan S. H. Rahemi M. Martinez-Romero D. Guillen F. Valverde J. M. Zapata P. J. . (2007). Reduction of pomegranate chilling injury during storage after heat treatment, role of polyamines. Postharvest Biol. Technol. 44, 19–25. doi: 10.1016/j.postharvbio.2006.11.001

[B108] Miura K. Furumoto T. (2013). Cold signaling and cold response in plants. Int. J. Mol. Sci. 14, 5312–5337. doi: 10.3390/ijms14035312 23466881 PMC3634503

[B109] Moshelion M. Halperin O. Wallach R. Oren R. A. M. Way D. A. (2015). Role of aquaporins in determining transpiration and photosynthesis in water-stressed plants, crop water-use efficiency, growth and yield. Plant Cell Environ. 38, 1785–1793. doi: 10.1111/pce.12410 25039365

[B110] Nair S. Singh Z. (2004). Chilling injury in mango fruit in relation to biosynthesis of free polyamines. J. Hortic. Sci. Biotechnol. 79, 515–522. doi: 10.1080/14620316.2004.11511798

[B111] Nishiyama I. (1976). Male Sterility caused by cooling treatment at the young microspore stage in rice plants. Proc. Crop Sci. Soc 45, 254–262. doi: 10.1626/jcs.45.254

[B112] Osman M. B. Milan A. R. (2006). Mangosteen- Garcinia Mangostana; Southampton Centre for Underutilised Crops (Southampton, UK: University of Southampton) 2006.

[B113] Owens C. L. Thomashow M. F. Hancock J. F. Iezzoni A. F. (2002). CBF1 orthologs in sour cherry and strawberry and the heterologous expression of CBF1 in strawberry. J. Amer. Soc Hortic. Sci. 127, 489–494. doi: 10.21273/JASHS.127.4.489

[B114] Ozturk L. Demir Y. (2002). In Vivo Vitro protective role proline. Plant Growth Regul. 38, 259–264. doi: 10.1023/A:1021579713832

[B115] Palta J. P. (1990). Stress interactions at the cellular and membrane levels. Hort Sci. 25, 1377–1381. doi: 10.21273/HORTSCI.25.11.1377

[B116] Paquin R. Bolduc R. Zizka J. Pelletier G. Lechasseur P. (1989). Tolerance au gel et teneur en sucres et en proline du collet du fraisier (Fragaria ananassa duch.) durant l’hiver. Can. J. Plant Sci. 69, 945–954. doi: 10.4141/cjps89-11

[B117] Polashock J. J. Arora R. Peng Y. Dhananjay N. Rowland L. J. (2010). Functional identification of a blueberry CBF/DREB-like element associated with cold acclimation and freezing tolerance. J. Am. Soc Hortic. Sci. 135, 40–48. doi: 10.21273/JASHS.135.1.40

[B118] Pradhan S. Goswami A. K. Singh S. K. Prakash J. Goswami S. Chinnusamy V. . (2017). Physiological and biochemical alterations due to low temperature stress in papaya genotypes. Indian J. Hortic. 74, 491–497. doi: 10.5958/0974-0112.2017.00096.2

[B119] Pradhan S. Goswami A. K. Singh S. K. Prakash J. Goswami S. Chinnusamy V. . (2019). Low temperature stress induced physiological and biochemical alterations in papaya genotypes. S. Afr. J. Bot. 123, 133–141. doi: 10.1016/j.sajb.2019.02.004

[B120] Pradhan S. Goswami A. K. Singh S. K. Prakash J. Goswami S. Viswanathan C. . (2018). Growth, nutrient acquisition and physiological responses of papaya (Carica papaya) plants to controlled low temperature stress. Indian J. Agric. Sci. 88, 726–732.

[B121] Rachappanavar V. Padiyal A. Sharma J. K. Gupta S. K. (2022). Plant hormone-mediated stress regulation responses in fruit crops- a review. Sci. Hortic., 304, 1000302. doi: 10.1016/j.scienta.2022.11130

[B122] Rajashekar C. B. Zhou H. Marcum K. B. Prakash O. (1999). Glycine betaine accumulation and induction of cold tolerance in strawberry (Fragaria X ananassa duch.) plants. Plant Sci. 148, 175–183. doi: 10.1016/S0168-9452(99)00136-3

[B123] Ram M. (2005). Papaya (New Delhi: Indian Council of Agricultural Research), 78.

[B124] Rey F. Zacarías L. Rodrigo M. J. (2020). Carotenoids, vitamin c, and antioxidant capacity in the peel of mandarin fruit in relation to the susceptibility to chilling injury during postharvest cold storage. Antioxidants. 9 (12), 1296. doi: 10.3390/antiox9121296 33348913 PMC7766470

[B125] Rooy S. S.B. Salekdeh G. H. Ghabooli M. Gholami M. Karimi R. (2017). Cold-induced physiological and biochemical responses of three grapevine cultivars differing in cold tolerance. Acta Physiol. Plant 39, 1–13. doi: 10.1007/s11738-017-2561-z

[B126] Ruelland E. Vaultier M. N. Zachowski A. Hurry V. (2009). Cold signalling and cold acclimation in plants. Adv. Bot. Res. 49, 35–150. doi: 10.1016/S0065-2296(08)00602-2

[B127] Sakamoto A. Murata N. (2002). The role of glycine betaine in the protection of plants from stress, clues from transgenic plants. Plant Cell Environ. 25, 163–171. doi: 10.1046/j.0016-8025.2001.00790.x 11841661

[B128] Sayyari M. Babalar M. Kalantari S. Martinez-Romero D. Guillen F. Serrano M. . (2011). Vapour treatments with methyl salicylate or methyl jasmonate alleviated chilling injury and enhanced antioxidant potential during postharvest storage of pomegranates. Food Chem. 124, 964–970. doi: 10.1016/j.foodchem.2010.07.036

[B129] Sayyari M. Babalar M. Kalantari S. Serrano M. Valero D. (2009). Effect of salicylic acid treatment on reducing chilling injury in stored pomegranates. Postharvest Biol. Technol. 53, 152–154. doi: 10.1016/j.postharvbio.2009.03.005

[B130] Shin H. Min K. Arora R. (2018). Exogenous salicylic acid improves freezing tolerance of spinach (Spinacia oleracea l.) leaves. Cryobiology 81, 192–200. doi: 10.1016/j.cryobiol.2017.10.006 29061524

[B131] Singh A. Deka B. C. Prakash J. Patel R. K. Ojah H. (2010). Problems and prospects of papaya cultivation in northeastern states of India in Acta Hortic. vol. 851, 61–66. doi: 10.17660/ActaHortic.2010.851.6

[B132] Sirooeinejad B. Zamani Z. Fatahi M. R. (2020). Study of physiological and biochemical responses to freezing stress in pomegranate (Punica granatum l.) trees during acclimation and deaclimation cycle. J. Hortic. Sci. Biotechnol. 95, 341–355. doi: 10.1080/14620316.2019.1674699

[B133] Smillie R. M. (1979). “The useful chloroplast, a new approach for investigating chilling stress in plants,” in Low temperature stress in crop plants (New York: Academic Press), 187–202.

[B134] Soitamo A. J. Piippo M. Allahverdiyeva Y. Battchikova N. Aro E. M. (2008). Light has a specific role in modulating arabidopsis gene expression at low temperature. BMC Plant Biol. 8, 1–13. doi: 10.1186/1471-2229-8-13 18230142 PMC2253524

[B135] Song T. Li K. Wu T. Wang Y. Zhang X. Xu X. . (2019). Identification of new regulators through transcriptome analysis that regulate anthocyanin biosynthesis in apple leaves at low temperatures. PloS One 14, e0210672. doi: 10.1371/journal.pone.0210672 30695036 PMC6350969

[B136] Sreedharan S. Shekhawat U. K. Ganapathi T. R. (2013). Transgenic banana plants over expressing a native plasma membrane aquaporin MusaPIP1; 2 display high tolerance levels to different abiotic stresses. Plant Biotechnol. J. 11, 942–952. doi: 10.1111/pbi.12086 23745761

[B137] Steponkus P. L. (1984). Role of the plasma membrane in freezing injury and cold acclimation. Annu. Rev. Plant Physiol. 35, 543–584. doi: 10.1146/annurev.pp.35.060184.002551

[B138] Steponkus P. L. (1993). A contrast of the cryostability of the plasma membrane of winter rye and spring oat. Adv. Low-Temperature Biol. 2, 211–312.

[B139] Storey W. B. (1969). Pistillate papaya flower: a morphological anomaly. Science 163, 401–405. doi: 10.1126/science.163.3865.401 17730186

[B140] Stushnoff C. Remmele R. L. Jr. Essensee V. McNeil M. (1993). “Low temperature induced biochemical mechanisms, implications for cold acclimation and de-acclimation,” in Interacting stresses on plants in a changing climate (Berlin, Heidelberg: Springer), 647–657. doi: 10.1007/978-3-642-78533-7_42

[B141] Sun X. Rikkerink E. H. Jones W. T. Uversky V. N. (2013). Multifarious roles of intrinsic disorder in proteins illustrate its broad impact on plant biology. Plant Cell 25, 38–55. doi: 10.1105/tpc.112.106062 23362206 PMC3584547

[B142] Sun X. Zhao T. Gan S. Ren X. Fang L. Karungo S. K. . (2016). Ethylene positively regulates cold tolerance in grapevine by modulating the expression of ETHYLENE RESPONSE FACTOR 057. Scient. Rep. 6, 1–14. doi: 10.1038/srep24066 PMC4819186 27039848

[B143] Swaaij A. V. Jacobsen E. Feenstra W. J. (1985). Effect of cold hardening, wilting and exogenously applied proline on leaf proline content and frost tolerance of several genotypes of solanum. Physiol. Plant 64, 230–236. doi: 10.1111/j.1399-3054.1985.tb02341.x

[B144] Taiz L. Zeiger E. (2002). Plant physiology, second edition (Sunderland, Massachusetts: Sinauer Associates Publishers), 607.

[B145] Takuhara Y. Kobayashi M. Suzuki S. (2011). Low-temperature-induced transcription factors in grapevine enhance cold tolerance in transgenic arabidopsis plants. J. Plant Physiol. 168, 967–975. doi: 10.1016/j.jplph.2010.11.008 21185622

[B146] Tambussi E. A. Bartoli C. G. Guiamet J. J. Beltrano J. Araus J. L. (2004). Oxidative stress and photodamage at low temperatures in soybean (Glycine max l. merr.) leaves. Plant Sci. 167, 19–26. doi: 10.1016/j.plantsci.2004.02.018

[B147] Theocharis A. Clement C. Barka E. A. (2012). Physiological and molecular changes in plants grown at low temperatures. Planta 235, 1091–1105. doi: 10.1007/s00425-012-1641-y 22526498

[B148] Thomashow M. F. (1998). Role of cold-responsive genes in plant freezing tolerance. Plant Physiol. 118, 1–8. doi: 10.1104/pp.118.1.1 9733520 PMC1539187

[B149] Thomashow M. F. (2010). Molecular basis of plant cold acclimation: insights gained from studying the CBF cold response pathway. Plant Physiol. 154, 571–577. doi: 10.1104/pp.110.161794 20921187 PMC2948992

[B150] Tindall H. D. (1994). Rambutan cultivation (Rome: Food and Agriculture Organization of the United Nations. FAO), 978–9-25103-325-8.

[B151] UDPSM (2002). “Plant propagation and planting in uplands,” in Training manual for municipal extension staff (Davao, Philippines: Upland Development Programin Southern Mindanao).

[B152] Upendra K. Shekhawat S. Srinivas L. Ganapathi T. R. (2011). Musa DHN-1, a novel multiple stress-inducible SK sub 3-type dehydrin gene, contributes affirmatively to drought-and salt-stress tolerance in banana. Planta 234, 915. doi: 10.1007/s00425-011-1455-3 21671068

[B153] Van der Paal J. Neyts E. C. Verlackt C. Bogaerts A. (2016). Effect of lipid peroxidation on membrane permeability of cancer and normal cells subjected to oxidative stress. Chem. Sci. 7, 489–498. doi: 10.1039/C5SC02311D 28791102 PMC5518669

[B154] Verheij E. W. M. Coronel R. E. (1992). “Edible fruits and nuts,” in Plant resources of south-East Asia, vol. 2. (Bogor: Prosea Foundation), 128–131.

[B155] Viktorova M. K. (1983). Plant growing in the tropics and subtropics (Moscow: Mir Publisher), 214–218.

[B156] Wang H. Blakeslee J. J. Jones M. L. Chapin L. J. Dami I. E. (2018). Exogenous abscisic acid enhances physiological, metabolic, and transcriptional cold acclimation responses in greenhouse-grown grapevines. Plant Sci. 293, 110437. doi: 10.1016/j.plantsci.2020.110437 32081274

[B157] Waraich E. A. Ahmad R. Ashraf M. Y. Saifullah Ahmad M. (2011). Improving agricultural water use efficiency by nutrient management in crop plants. Acta Agric. Scand. B Soil Plant Sci. 61, 291–304. doi: 10.1080/09064710.2010.491954

[B158] Waraich E. A. Ahmad R. Halim A. Aziz T. (2012). Alleviation of temperature stress by nutrient management in crop plants, a review. J. Soil Sci. Plant Nutt. 12, 221–244. doi: 10.4067/S0718-95162012000200003

[B159] Wei Q. Ma Q. Ma Z. Zhou G. Feng F. Le S. . (2019). Genome-wide identification and characterization of sweet orange (Citrus sinensis) aquaporin genes and their expression in two citrus cultivars differing in drought tolerance. Tree Genet. Genomes 15, 17. doi: 10.1007/s11295-019-1321-1

[B160] White M. A. Diffenbaugh N. S. Jones G. V. Pal J. S. Giorgi F. (2006). Extreme heat reduces and shifts united states premium wine production in the 21st century. Proc. Natl. Acad. Sci. 103, 11217–11222. doi: 10.1073/pnas.060323010 16840557 PMC1544068

[B161] Wilkinson S. Clephan A. L. Davies W. J. (2001). Rapid low temperature-induced stomatal closure occurs in cold-tolerant cammelina communis leaves but not in cold-sensitive tobacco leaves, *via* a mechanism that involves apoplastic calcium but not abscisic acid. Plant Physiol. 126, 1566–1578. doi: 10.1104/pp.126.4.1566 11500555 PMC117156

[B162] Wisniewski M. Norelli J. Bassett C. Artlip T. Macarisin D. (2011). Ectopic expression of a novel peach (Prunus persica) CBF transcription factor in apple (Malus × domestica) results in short-day induced dormancy and increased cold hardiness. Planta 233, 971–983. doi: 10.1007/s00425-011-1358-3 21274560

[B163] Wisniewski M. Webb R. Balsamo R. Close T. J. Yu X. M. Griffith M. . (1999). Purification, immunolocalization, cryoprotective, and anti-freeze activity of PCA60, a dehydrin from peach (Prunus persica). Physiol. Plant 105, 600–608. doi: 10.1034/j.1399-3054.1999.105402.x

[B164] Xiao H. Tattersall E. A. Siddiqua M. K. Cramer G. R. Nassuth A. (2008). CBF4 is a unique member of the CBF transcription factor family of vitis vinifera and vitis riparia. Plant Cell Environ. 31, 1–10. doi: 10.1111/j.1365-3040.2007.01741.x 17971068

[B165] Xu Y. Hu W. Liu J. Song S. Hou X. Jia C. . (2020). An aquaporin gene MaPIP2-7 is involved in tolerance to drought, cold and salt stresses in transgenic banana (Musa acuminata l.). Plant Physiol. Biochem. 147, 66–76. doi: 10.1016/j.plaphy.2019.12.011 31841963

[B166] Xu Y. Liu J. Jia C. Hu W. Song S. Xu B. . (2021). Overexpression of a banana aquaporin gene MaPIP1; 1 enhances tolerance to multiple abiotic stresses in transgenic banana and analysis of its interacting transcription factors. Front. Plant Sci. 12. doi: 10.3389/fpls.2021.699230 PMC8424054 34512687

[B167] Xu C. Wang M. T. Yang Z. Q. Zheng Q. T. (2020). Low temperature and low irradiation induced irreversible damage of strawberry seedlings. Photosynthetica 58, 156–164. doi: 10.32615/ps.2020.001

[B168] Yadav S. K. (2010). Cold stress tolerance mechanisms in plants. A review. Agron. Sustain. Dev. 30, 515–527. doi: 10.1051/agro/2009050

[B169] Yang M. T. Chen S. L. Lin C. Y. Chen Y. M. (2005). Chilling stress suppresses chloroplast development and nuclear gene expression in leaves of mung bean seedlings. Planta 221, 374–385. doi: 10.1007/s00425-004-1451-y 15599759

[B170] Yang W. Liu X. D. Chi X. J. Wu C. A. Li Y. Z. Song L. L. . (2011). Dwarf apple MbDREB1 enhances plant tolerance to low temperature, drought, and salt stress *via* both ABA-dependent and ABA-independent pathways. Planta 233, 219–229. doi: 10.1007/s00425-010-1279-6 20967459

[B171] Yang Y. He M. Zhu Z. Li S. Xu Y. Zhang C. . (2012). Identification of the dehydrin gene family from grapevine species and analysis of their responsiveness to various forms of abiotic and biotic stress. BMC Plant Biol. 12, 1–17. doi: 10.1186/1471-2229-12-140 22882870 PMC3460772

[B172] Yelenosky G. (1985). Cold hardiness in citrus. Hortic. Rev. 7, 201–238. doi: 10.1002/9781118060735.ch5

[B173] Yokoi S. Higashi S. I. Kishitani S. Murata N. Toriyama K. (1998). Introduction of the cDNA for shape arabidopsis glycerol-3-phosphate acyltransferase (GPAT) confers unsaturation of fatty acids and chilling tolerance of photosynthesis on rice. Mol. Breed. 4, 269–275. doi: 10.1023/A:1009671231614

[B174] Yooyongwech S. Sugaya S. Sekozawa Y. Gemma H. (2009). Differential adaptation of high-and low-chill dormant peaches in winter through aquaporin gene expression and soluble sugar content. Plant Cell Rep. 28, 1709. doi: 10.1007/s00299-009-0770-7 19760270

[B175] Yuan W. Liu J. Takac T. Chen H. Li X. Meng J. . (2021). Genome-wide identification of banana csl gene family and their different responses to low temperature between chilling-sensitive and tolerant cultivars. Plants 10, 122. doi: 10.3390/plants10010122 33435621 PMC7827608

[B176] Yun K. Y. Park M. R. Mohanty B. Herath V. Xu F. Mauleon R. . (2010). Transcriptional regulatory network triggered by oxidative signals configures the early response mechanisms of japonica rice to chilling stress. BMC Plant Biol. 10, 16. doi: 10.1186/1471-2229-10-16 20100339 PMC2826336

[B177] Zareei E. Karami F. Gholami M. Ershadi A. Avestan S. Aryal R. . (2021). Physiological and biochemical responses of strawberry crown and leaf tissues to freezing stress. BMC Plant Biol. 21, 1–17. doi: 10.1186/s12870-021-03300-2 34773991 PMC8590311

[B178] Zhang W. Jiang H. Cao J. Jiang W. (2021). Advances in biochemical mechanisms and control technologies to treat chilling injury in postharvest fruits and vegetables. Trends Food Sci. Technol. 113, 355–365. doi: 10.1016/j.tifs.2021.05.009

[B179] Zhang C. K. Lang P. Dane F. Ebel R. C. Singh N. K. Locy R. D. . (2005). Cold acclimation induced genes of trifoliate orange (Poncirus trifoliata). Plant Cell Rep. 23, 764–769. doi: 10.1007/s00299-004-0883-y 15449021

[B180] Zhang Y. Tang H. R. Luo Y. Hou Y. X. (2009). Responses of antioxidant enzymes and compounds in strawberry (Fragaria × ananassa ‘Toyonaka’) to cold stress. New Z. J. Crop Hortic. Sci. 37, 383–390. doi: 10.1080/01140671.2009.9687594

[B181] Zhou H. W. Dong L. Ben-Arie R. Lurie S. (2001). The role of ethylene in the prevention of chilling injury in nectarines. J. Plant Physiol. 158, 55–61. doi: 10.1078/0176-1617-00126

[B182] Zhu J. Dong C. H. Zhu J. K. (2007). Interplay between cold-responsive gene regulation, metabolism and RNA processing during plant cold acclimation. Curr. Opin. Plant Biol. 10, 290–295. doi: 10.1016/j.pbi.2007.04.010 17468037

[B183] Zinta G. Singh R. K. Kumar R. (2022). Cold adaptation strategies in plants-an emerging role of epigenetics and antifreeze proteins to engineer cold resilient plants. Front. Genet. 13. doi: 10.3389/fgene.2022.909007 PMC9459425 36092945

[B184] Zuther E. Schaarschmidt S. Fischer A. Erban A. Pagter M. Mubeen U. . (2019). Molecular signatures associated with increased freezing tolerance due to low temperature memory in arabidopsis. Plant Cell Environm. 42, 854–873. doi: 10.1111/pce.13502 30548618

